# M1-derived extracellular vesicles polarize recipient macrophages into M2-like macrophages and alter skeletal muscle homeostasis in a hyper-glucose environment

**DOI:** 10.1186/s12964-024-01560-7

**Published:** 2024-03-27

**Authors:** Stefano Tacconi, Francesco Vari, Carolina Sbarigia, Diana Vardanyan, Serena Longo, Francesco Mura, Federica Angilè, Audrey Jalabert, Ferninand Blangero, Assia Eljaafari, Laurence Canaple, Daniele Vergara, Francesco Paolo Fanizzi, Marco Rossi, Claire Crola Da Silva, Elizabeth Errazuriz-Cerda, Christel Cassin, Rienk Nieuwland, Anna Maria Giudetti, Sophie Rome, Luciana Dini

**Affiliations:** 1 CarMeN Laboratory, UMR (INSERM 1060/INRA 1397), HCL, Lyon 1 University, Pierre- Bénite, France; 2 Department of Biology and Biotechnology “C. Darwin”, Sapienza University of Roma, Roma, Italy; 3 Department of Biological and Environmental Sciences and Technologies (Di.S.Te.B.A.), University of Salento, Lecce, Italy; 4 Research Center of Nanotechnologies for Engineering (CNIS), Sapienza University of Roma, Roma, Italy; 5 Ecole Normale Supérieure de Lyon, SFR BIOSCIENCES UAR3444, Lyon, France; 6 Department of Basic and Applied Sciences for Engineering, University of Rome Sapienza, Roma, Italy; 7 Centre d’Imagerie Quantitative Lyon Est (CIQLE), Lyon 1 University, Lyon, France; 8 Laboratory of Experimental Clinical Chemistry, Department of Clinical Chemistry, Amsterdam Vesicle Center, AMC, Amsterdam UMC, Amsterdam, Netherlands

**Keywords:** Extracellular vesicles, Hyperglycemia, Macrophage, Lipid metabolism, Skeletal muscle, Oxidative phosphorylation

## Abstract

**Background:**

Macrophages release not only cytokines but also extracellular vesicles (EVs). which are small membrane-derived nanovesicles with virus-like properties transferring cellular material between cells. Until now, the consequences of macrophage plasticity on the release and the composition of EVs have been poorly explored. In this study, we determined the impact of high-glucose (HG) concentrations on macrophage metabolism, and characterized their derived-EV subpopulations. Finally, we determined whether HG-treated macrophage-derived EVs participate in immune responses and in metabolic alterations of skeletal muscle cells.

**Methods:**

THP1-macrophages were treated with 15mM (MG15) or 30mM (MG30) glucose. Then, M1/M2 canonical markers, pro- and anti-inflammatory cytokines, activities of proteins involved in glycolysis or oxidative phosphorylation were evaluated. Macrophage-derived EVs were characterized by TEM, NTA, MRSP, and ^1^H-Nuclear magnetic resonance spectroscopy for lipid composition. Macrophages or C2C12 muscle cells were used as recipients of MG15 and MG30-derived EVs. The lipid profiles of recipient cells were determined, as well as proteins and mRNA levels of relevant genes for macrophage polarization or muscle metabolism.

**Results:**

Untreated macrophages released small and large EVs (sEVs, lEVs) with different lipid distributions. Proportionally to the glucose concentration, glycolysis was induced in macrophages, associated to mitochondrial dysfunction, triacylglycerol and cholesterol accumulation. In addition, MG15 and MG30 macrophages had increased level of CD86 and increase release of pro-inflammatory cytokines. HG also affected macrophage sphingolipid and phospholipid compositions. The differences in the lipid profiles between sEVs and lEVs were abolished and reflected the lipid alterations in MG15 and MG30 macrophages. Interestingly, MG15 and MG30 macrophages EVs induced the expression of CD163, Il-10 and increased the contents of triacylglycerol and cholesterol in recipient macrophages. MG15 lEVs and sEVs induced insulin-induced AKT hyper-phosphorylation and accumulation of triacylglycerol in myotubes, a state observed in pre-diabetes. Conversely, MG30 lEVs and sEVs induced insulin-resistance in myotubes.

**Conclusions:**

As inflammation involves first M1 macrophages, then the activation of M2 macrophages to resolve inflammation, this study demonstrates that the dialog between macrophages through the EV route is an intrinsic part of the inflammatory response. In a hyperglycemic context, EV macrophages could participate in the development of muscle insulin-resistance and chronic inflammation.

**Supplementary Information:**

The online version contains supplementary material available at 10.1186/s12964-024-01560-7.

## Background

Extracellular vesicles (EVs) are lipid-derived vesicles released from cells that convey proteins, lipids, nucleic acids, and other metabolites among different cell types. All cells release EVs from the plasma membrane (PM) whereas eukaryotic cells also release EVs called ‘exosomes’ formed during the inward budding of the limiting membrane of the late endosomes called ‘multivesicular bodies’ (MVBs) [1]. Some subsets of MVBs can fuse with the plasma membrane and release their vesicular cargoes outside the cells. As the separation of these subtypes of EVs based on their composition is until now not definitively resolved, the International Society for Extracellular Vesicles (ISEV) recommends the terminology of large EVs (lEVs, > 200 nm) to consider EVs originated from PM, and small EVs (sEVs, < 200 nm) to consider EVs released from the endolysosomal pathway or small PM buddings [2, 3]. Until now, this nomenclature has permitted to identify subtypes of EVs with specific lipid, RNA, and protein compositions [4–7] confirming that this nomenclature is indeed able to discriminate EV subpopulations likely originating from different cellular compartment. Although poorly studied, data on adipocyte-derived EVs [4] or on skeletal muscle-derived EVs [8] indicate that lEVs and sEVs might have specific biological functions.

Surprisingly, although immune cells are the largest purveyor of EVs in the blood, the respective functions of lEVs and sEVs released from immune cells are poorly understood. In addition, although immune cell-derived EVs are now recognized as important actors of the immune system [9, 10], the impact of the immune cell environment on the release of lEVs and sEVs is also unknown. Nevertheless, two studies demonstrated that in response to cytokines [11] or endotoxins [12] immune cells modulate their metabolism to secrete pro- or anti-inflammatory mediators and had increased release of EVs. Among immune cells, macrophages are key cells of innate immunity that participate also in tissue remodeling. Therefore, macrophages have a pleiotropic role within tissues to maintain tissue homeostasis in health and diseases. To fulfill this role, they must adapt to their microenvironment which results in the production of different macrophage phenotypes. Until now the consequences of macrophage plasticity on the release and composition of EVs have been explored in the fields of cancer, infections, and tissue repair [13–17]. But of importance are the modifications of the macrophage nutritional environment. Indeed, lipid metabolism plays a major role in the activation of both M1 and M2 macrophages, *e.g*.; fatty acid oxidation is essential for the activation of inflammasome in M2 macrophages, and glycolysis fuels fatty acid oxidation in M1 macrophages [18]. Therefore metabolic alterations such as those obtained during the development of high-fat diet-induced obesity or associated with alteration of glucose homeostasis (*i.e*.; diabetes) are associated with the development of low-grade inflammation and macrophage infiltration and polarization inside metabolic tissues [19]. Indeed, it was shown that under high-glucose (HG) conditions M1 macrophages exhibited an amplified pro-inflammatory response and had decreased production of anti-inflammatory mediators leading to sustained inflammation [20]. During diabetes onset and progression, hyperglycemia increased ROS production in macrophages, which favor induction of M1 pro-inflammatory macrophages [20]. Therefore, understanding the contribution of macrophages in chronic inflammation under HG concentrations is vital for developing targeted therapeutic strategies that can modulate macrophage function and mitigate the detrimental effects of hyperglycemia on inflammatory processes. In that context, it was found that a high-glucose (HG) treatment induced the release of sEVs from macrophages (HG-sEVs) [21]. HG-sEVs induced activation and proliferation of mesangial cells, and secretion of extracellular matrix and inflammatory cytokines, compared to control-sEVs. HG-sEVs activated mesangial cells through the transfer of TGF-β1 mRNA. This study has demonstrated that a state of hyperglycemia may modify the release and the function of sEVs from macrophages which consequently participate in the progression of diabetic nephropathy [21]. The same group also demonstrated that HG-sEVs contained an increased concentration of miR-7002-5p which induced dysfunction, autophagy inhibition, and inflammation in recipient mouse tubular epithelial cells or kidney [22]. Taken together, these 2 studies have provided a proof-of-concept that glucose itself can modulate the release of extracellular vesicles from macrophages.

However, the functions of the lEVs and sEVs released from macrophages are unknown, and the consequences of the glucose concentration on macrophage metabolism underlying the modulation of EV release have not yet been determined. As EVs are mainly lipid-derived nanovesicles, we hypothesized that the modulation of EV release from macrophages in response to glucose may be associated with alterations in macrophage lipid metabolism. Therefore, in this paper, we have determined the impact of HG concentrations on macrophage phenotype and metabolism. Then we studied how these lipidomic alterations can affect the lipid composition of macrophage sEVs and lEVs. Finally, we determined whether lEVs and sEVs could participate in macrophage cross-talks during the development of the inflammatory response and in the alteration of skeletal muscle (SkM) homeostasis, a tissue known to be infiltrated by macrophages in a hyperglycemic condition such as obesity [23, 24]. Indeed, macrophages are important actors in the regeneration and maintenance of muscle homeostasis through their release of growth factors and cytokines that regulate muscle satellite cells and myofibroblast activation [25, 26]. In this study we demonstrated an unspected role of macrophage-derived EVs during inflammation and highligh their contributions to the regulation of muscle homeostasis in situation of hyperglycemia.

## Methods

### Cell culture conditions and treatments

Human THP-1 monocytes were grown in RPMI-1640 (Cambrex, NJ) containing 2 mM L-glutamine, 10 mM HEPES, 1 mM sodium pyruvate, 1500 mg/L sodium bicarbonate and 2000 mg/L glucose (11mM) and supplemented with 10% heat-inactivated Fetal Bovine Serum (FBS) (Cambrex, NJ), 2mM L-glutamine (Cambrex), 100IU/mL penicillin and streptomycin and 10,000U/mL amphotericin (antimycotic solution) (Sigma Aldrich, MA). At the concentration of 5 × 10^5^ cells/mL, monocytes were differentiated into macrophages with 100ng/mL Phorbol 12-Myristate 13-Acetate (PMA) as in [27]. After 72 h differentiation, macrophages were treated either with 15mM (MG15) or 30mM glucose (MG30) (final concentrations) for 24 h. As a positive control, macrophages were polarized into M1 macrophages with 100ng/mL lipopolysaccharides (LPS, Sigma Aldrich, MA) and 10ng/mL IFNγ (ThermoFisher Scientific, MA), or into M2 macrophages with 10ng/mL IL-4 for 24 h. As a control of osmotic stress, macrophages were treated with 30mM mannitol (final concentration).

Mouse myoblasts C2C12 cells (from ATCC® CRL-1772™) were routinely maintained in DMEM 4500 mg/L glucose supplemented with 10% heat-inactivated FBS, 1000UI/mL penicillin, 1000UI/mL streptomycin, and 2mM L-glutamine (37 °C, 5% CO_2_). At confluence, differentiation was induced with DMEM 4.5 g/L glucose supplemented with 2% Horse Serum, for one week.

To assess the effects of lEVs and sEVs, on recipient macrophages, 2 µg/mL of EVs were added to 1 × 10^6^ THP1 macrophages or to C2C12 myotubes grown in EV-depleted medium. After EV treatment, myotubes were serum-starved for 4 h in DMEM 1.5 g/L glucose, then were treated with 100nM insulin (Sigma-Aldrich, MA) for 10 min, and immediately lysed for protein extraction.

### EV isolation and sizing

To removed any particles from FBS, RPMI was centrifuged at 100,000 g (4 °C, overnight). The supernatant was filtered at 0.2 μm before use. For each experimental group, 6 T75 flasks containing each 10 × 10^6^ differentiated THP-1 were grown in 20 ml of EV-depleted medium for 24 h. We have validated that this EV-depleted medium did not affect macrophage viability (Additional Fig. 1A). EVs purification was realized by differential centrifugation: 500 g (10 min, RT), the resulting supernatant was centrifuged at 800 g (10 min, RT) and then at 2,000 g (20 min, RT). To pellet lEVs, the resulting supernatant was centrifuged at 20,000 g (30 min, 4 °C). To pellet sEVs, the supernatant was filtered through a 0.22 μm filter (polyethersulfone membrane filter units, Thermo Fisher Scientific, MA) and then centrifuged at 100,000 g (70 min, 4 °C, 50.2 Ti fixed-angle rotor (Beckman Coulter)) and rinsed with 26 ml PBS without calcium and magnesium, previously filtered at 0.1 μm. The resulting pellets of lEVs or sEVs were resuspended in the same PBS (50µL). EV concentrations and sizes were determined by nanotracking analyses (NTA) (sample dilution 1/50, total number of frames: 1438, camera level: 16, detection threshold: 4, 3x videos of 60s). As shown on Additional Fig. 1B, we have determined the quantity of particles remained in EV-depleted medium by Microfluidic Resistive Pulse Sensing (MRPS) as in [28, 29]. In this experiment, the same volume of 24 h conditioned medium, with or without macrophages, was used to purify EVs. The data showed that the EV-depleted medium contained a very small amount of remaining particules that can’t be removed and which represented less than 10% of the sEV pellet. Among these particles, residual lipoproteins from FBS could be detected (Additional Fig. 1C). We also validated that the high-glucose treatment did not affect macrophage viability and thus did not induce the release of apoptotic bodies (Additional Fig. 1D).

### Cell viability assay

3-[4,5-dimethylthiazol-2-yl]-2,5 diphenyl tetrazolium bromide (MTT) assay was performed according to [30]. Untreated, MG15 and MG30 macrophages were incubated with 1 mg/mL MTT in RPMI-1640 (2 h, 37 °C and 5% CO2). Cells were then washed 3x in PBS (0.2 M, pH 7.4) and the reduced MTT formazan crystals were solubilized with dimethyl sulfoxide (DMSO) (Carlo Erba, IT). Absorbance was measured at 570 nm (Ultrospec 4000 UV/Visible Spectrophotometer, Pharmacia Biotech, IT).

### Scanning Electron Microscopy (SEM)

Untreated, MG15 and MG30 macrophages grown on glass coverslips were fixed with 2.5% glutaraldehyde in 0.1 mol/L cacodylate buffer (pH 7.4, 1 h, 4 °C) and post-fixed with 1% OsO4 in the same buffer. Cells were dehydrated with acetone (25%, 50%, 70%, 90% and 100%) followed by Critical Point Dryer CPD EMITECH K850 (Quorum Technologies Ltd, UK). Stub-mounted specimens were gold-coated with a Balzers Union SCD 040 (Balzers Union, LI) and examined with a Zeiss EVO HD 15 scanning microscope (Ziess, DE).

### Transmission Electron Microscopy (TEM)

Cells were fixed and dehydrated as for SEM and embedded in Spurr resin. 60 nm sections were examined under a transmission electron microscope JEOL 1400JEM (Tokyo, Japan) operating at 80 kV equipped with a camera Orius 600 gatan and Digital Micrograph (Product version: 1.7). After isolation, EVs were fixed with 0.1% of paraformaldehyde in PBS (30 min, RT). Fixed EVs were stained with 2% uranyl acetate (7 min, RT), loaded on formvar-carbon coated grids and observed with a Zeiss Auriga Scanning Electron Microscope (Zeiss, DE) equipped with the STEM module operating at 20 kV. Immunogold labelling to detect CD63 and CD81 at the surface of extracellular vesicles was realized as in [31].

### Western blot analysis

Cells were lysed in RIPA buffer (NaCl 150mM, Tris-HCl 50mM pH 8, MgCl2 2mM, SDS 0.1%, Deoxycholic Acid 0.5%, NP40 1%) containing phenylmethylsulfonyl fluoride and protease inhibitor cocktail (ThermoFisher Scientific, MA). After 5 min sonication, insoluble material was pelleted (10 min, 13,000 g, 4 °C). Supernatants were transferred into a new tube, and protein concentration was measured with the Bradford method. 15–30 µg of proteins, or 10 µg EV proteins were separated by sodium dodecylsulfate-polyacrylamide gel electrophoresis (10–12% polyacrylamide, SureCast™ Acrylamide Solution, ThermoFisher Scientific, MA) and transferred onto PVDF membranes. Membranes were blocked with 5% nonfat dry milk and/or 3% bovine serum albumin (BSA) in Tris-buffered saline containing 0.1% Tween20 (TTBS) (1 h, RT). After overnight incubation with primary antibodies (Additional Table 1A), membranes were washed with TTBS, incubated with horseradish peroxidase-conjugated secondary antibodies diluted in TTBS in 5% nonfat dry milk and/or 3% BSA (Additional Table 1A). Immunoreactive bands were detected by using enhanced chemiluminescence (ECL) reagent (Immobilon Crescendo Western HRP substrate; Merck Millipore, DE). Band densities were quantified by densitometry using Image LabTM Version 6.0.1 2017 (Bio-Rad Laboratories, CA).

### Enzyme- linked immunosorbent assays (ELISA) for cytokines quantification

All products are from the DuoSet ELISA Ancillary Reagent Kit 2 (Cat#DY008B, R&D System, MN). Specific cytokine antibodies were diluted in the Plate-Coating Buffer and coated in microplates overnight. Pre-coated microplates were pre-treated for 1 h with a blockade reagent and rinced. Macrophages conditioned medium was loaded in the pre-coated microplates and incubated for 2 h (*n* = 3 biological replicates and *n* = 2 technical replicates). After a step of washing, Streptavidin-HRP (Cat#DY998, R&D System, MN) was added for 30 min followed by the addition of TMB Substrate. The reaction was stopped by adding the stop solution. Cytokine concentrations were determined using a Spectrophotometer (Multiskan GO, 595 nm). Between each steps, wells were washed 4 times with the washing buffer.

### Real-time PCR

Total RNA was extracted using Trizol (Invitrogen, USA). 1.5 µg of RNA was converted into cDNA using a single-step SuperScriptTM IV kit (Invitrogen, Carlsbad, CA) protocol. PCR was realized by using CFX ConnectTM Real-Time PCR Detection System (BIORAD, Hercules, CA). Primer sequences are in Additional Table 1B.

### Oil Red-O lipid droplet staining

Post EV treatment, cells were grown on 11 mm glass coverslips, washed with DPBS, fixed 5 min with 10% formalin, washed 5 min with 60% isopropanol and left until dry. Subsequently, cells were incubated for 20 min in 0.5% Oil Red-O/isopropyl alcohol and washed several times with DPBS. Then nuclei were stained with 1µM diamidino-2-phenylindole (DAPI). The coverslips were washed and visualized with a Zeiss Microscope equipped with Apotome (Zeiss, Germany). Images analysis and lipid droplet quantifications were performed as reported in [32].

### Extracellular lactate measurement

The levels of lactate in the conditioned medium of untreated macrophages, MG15, and MG30 macrophages were measured as reported in [33].

### Bioenergetic measurements using the Seahorse analyzer

Oxygen consumption rate (OCR, pmoles/min) were measured using a Seahorse XFe 24 metabolic flux analyzer (Agilent Technologies, CA). Cells were plated in a Seahorse 24-well cell culture microplate at a density of 105 cells per well. Cells were equilibrated for 1 h in DMEM pH7.4 (2mM glutamine, 2mM sodium pyruvate, 10mM glucose) at 37°c in a CO2-free incubator. Mitochondrial stress test was performed using the following concentrations of sequentially injected compounds: 1.5µM mitochondrial ATP synthase inhibitor oligomycin, 1µM mitochondrial uncoupler (carbonylcyanide-p-trifluoromethoxyphenylhydrazone, FCCP) and 1 μm a mix of mitochondrial respiratory chain inhibitors rotenone/antimycin. The OCR was monitored under basal conditions and after addition of each compound, with 3 measurements of OCR for a period of 3 min. Measurements were normalized by the number of nuclei/well (Hoechst-stained nuclei imaged with Cytation 1 Cell Imaging Reader (BioTek, Agilent Technologies, CA).

### Activities of hexokinase (HK) and AcylCoA:diacylglycerol acyltransferase (DGAT)

HK activity was measured spectrophotometrically following the reduction of NADP^+^ at 340 nm. The reaction mixture contained 40mM Tris, 22mM Mg-acetate, 10mM β-mercaptoethanol, (pH 8.5), 0.75mM NADP^+^, 10 mM ATP, and 1.0 IU glucose-6‐phosphate dehydrogenase. The reaction was induced with 5 mM glucose. DGAT activity was measured using endogenous diacylglycerols as substrates, in digitonin-permeabilized macrophages, in the presence of [1-^14^C]palmitoyl-CoA (240 Bq/mol) as reported in [34]. The incubation was stopped after 90 s by the addition of 2mL of methanol: chloroform (2:1, v/v). After lipid extraction, TG were isolated by thin-layer chromatography on Silica G as reported in [34]. The silica, containing the TG fraction, was scraped from the plate and radioactivity was measured.

### Thin-layer chromatography (TLC)

Lipids were extracted by the Bligh and Dyer procedure. An equal amount mg of protein per sample was loaded onto a silica gel plates and separated by thin-layer chromatography (TLC). Plates were developed with hexane/ethyl ether/acetic acid (70/30/1; v/v/v) for neutral lipids, with chloroform/methanol/water (65/25/4; v/v/v) for phospholipids and with toluene/methanol (70:30; v/v) for sphingolipids. Then, plates were uniformly sprayed with 10% cupric sulfate in 8% aqueous phosphoric acid, allowed to dry (10 min, RT) and placed at 145 °C, 10 min. Identification of different species was made by developing specific standards with the same experimental conditions. The quantification of the spots was carried out by densitometry with ChemiDoc MP imager in white light epi-illumination (590/110 Filter), after coloration of the same and carbonization. Spot intensity was measured by densitometric analysis using Image Lab Version 6.1 (Bio-Rad Laboratories, Inc., Segrate (MI), Italy) software. The variability in the position of the spots on the various plates can depend on the run time. However, the RF (the ratio between the fronts), which remains constant, allows the individual lipid species to be properly identified by comparing them with the RF of standard lipids. The values of the individual lipid species were compared to the total number of lipid species present in the same sample, therefore expressed as a percent of the total. Then, the comparison was made on the percentage of expression of each lipid species in the sample, with that of the same lipid species in other samples. This semi-quantitative method eliminates the bias of loading different quantities of samples.

### ^1^H-Nuclear magnetic resonance (NMR) Spectroscopy

All measurements were performed on a Bruker Avance III 600 Ascend NMR spectrometer (Bruker, Biospin Milan, IT) operating at 600.13 MHz for 1 h observation, equipped with a TCI cryoprobe incorporating a z-axis gradient coil and automatic tuning-matching. Experiments were acquired at 300 K in automation mode after loading individual samples on a Bruker Automatic Sample Changer, interfaced with the software IconNMR (Bruker). Lipid extracts were dissolved in 600 µL of CD_3_OD/CDCl_3_ (1:2 mix) and transferred into a 5 mm NMR tube, using tetramethylsilane (TMS, δ = 0.00ppm) as internal standard. A one-dimensional experiment (zg Bruker pulse program) was performed with 256 scans, 64 K data points, a spectral width of 20.0276ppm, 2s delay, p1 8µs, and 2.73 acquisition time. The resulting FIDs were multiplied by an exponential weighting function corresponding to a line broadening of 0.3 Hz before Fourier transformation, automated phasing, and baseline correction. Lipid species were assigned based on 2D NMR spectra analysis (2D ^1^H Jres, ^1^H COSY, ^1^H-13 C HSQC) and comparison with published data [35].

### Statistical analyses

Data are expressed as Means ± SD. Multiple comparisons were performed by two-way ANOVA. Comparisons between two groups were performed using a student’s *t*-test (GraphPad Prism 7 software, GraphPad Software, San Diego, CA). *p* < 0.05 were considered significant vs. untreated.

## Results

### Hyper-glucose (HG) up-regulated markers of polarization and cytokine release associated with macrophage M1 phenotype and altered macrophage lipid metabolism

**Fig. 1:**
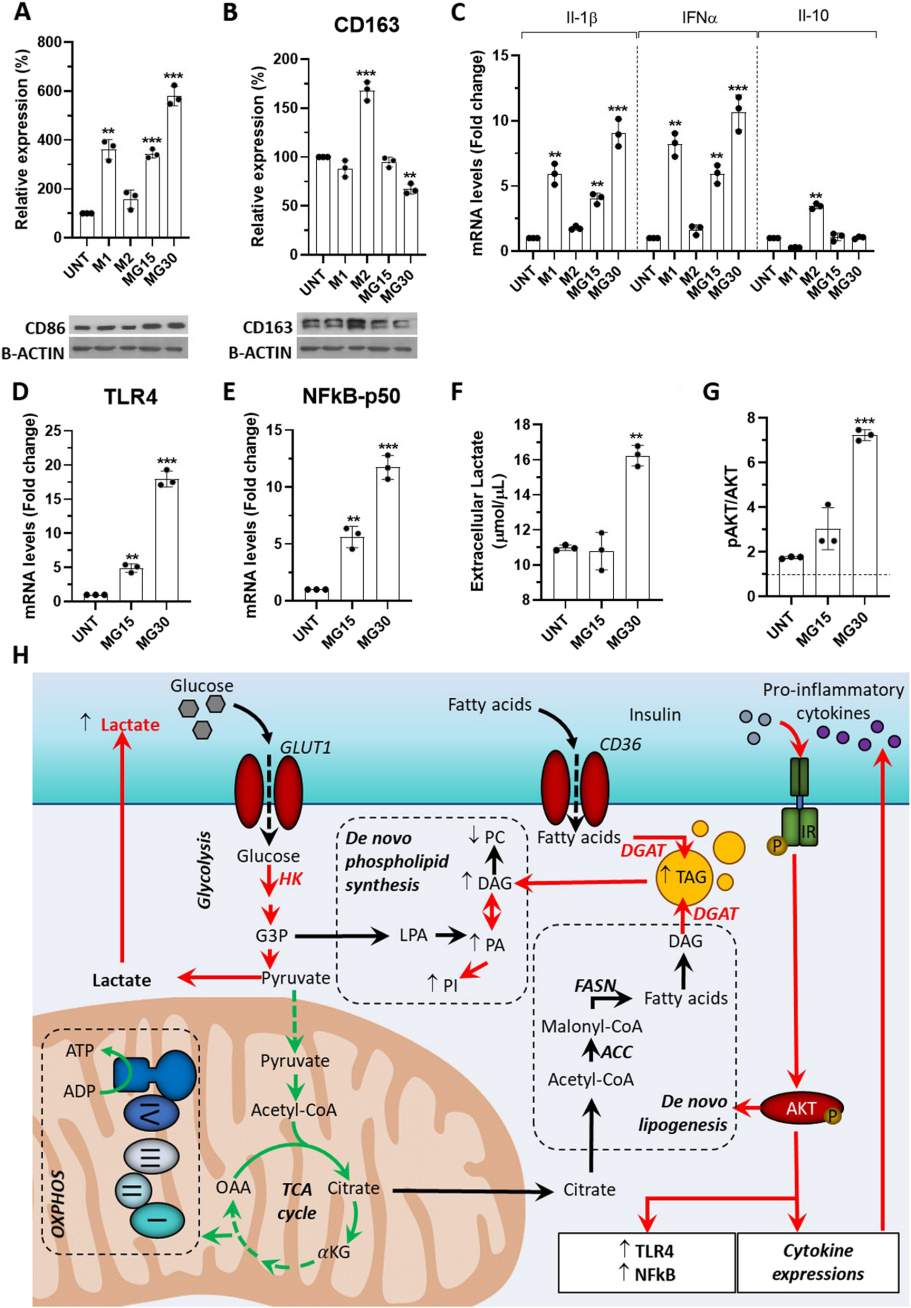
High-glucose induced M1 markers and cytokine production in macrophages. **A-B **Protein levels of CD86 and CD163 determined by WB. UNT=untreated macrophages, MG15= 15mM glucose, MG30= 30mM glucose. **C** mRNA levels of Il-1β, IFNα and Il-10. M1=THP-1-derived macrophages treated with IFNγ and lipopolysaccharide (LPS) were used as a positive control of M1 polarization, M2=THP-1-derived macrophages treated with IL-4, used as a positive control of M2 polarization. **D** toll-like receptor 4 (TLR4) and (**E**) subunit p-50-nuclear factor kappa-light-chain-enhancer of activated B cells (p50-NFkB) in macrophages. All RT-PCR data normalized to glyceraldehyde 3-phosphate dehydrogenase (GAPDH) mRNA level, then expressed as fold changes of untreated macrophages. **F** Lactate concentration in the macrophage-conditioned medium. **G** WB of phospho AKT in response to insulin, normalized to total AKT. **H** Summary of the effect of glucose treatement on macrophage lipid metabolism. In red, the reactions that are up-regulated and in green, the reactions that are down-regulated, in a glucose dependent manner, and validated in this study values are means ± SD (*n* = 3); *p* values are from student *t*-test (treated *vs* untreated), (*)* p*<0.05, (**) *p*<0.01, (***) *p*<0.001. ACC: acetyl-CoA carboxylase; aKG: α-ketoglutaric acid; AKT: protein kinase B; ADP: adenosine diphosphate; ATP: adenosine triphosphate; CD36: Cluster Determinant 36; DAG: diacylglycerol; DGAT: diacylglycerol acyltransferase 1/2; FASN: fatty acid synthase; G3P: glyceraldehyde 3 phosphate; GLUT1: glucose transporter 1; HK: hexokinase; IR: insulin receptor; LPA: lysophosphatidic acid; OAA: oxaloacetic acid; PA: phosphatidic acid; PC: phosphatidylcholine; PI: phosphatidylinositol; TAG: triacylglycerol; TCA: tricarboxylic acid

To understand the effect of HG concentrations on the release of EVs from macrophages, we first characterized the phenotypes of HG-treated macrophages. Two high glucose concentrations were used, i.e.; 15mM (MG15 macrophages), to mimic a state of hyperglycemia, a risk factor for many diseases (e.g.; ischemic cardiovascular injuries [36], renal diseases [37], sepsis [38], cancer [39] and diabetes), and 30mM (MG30 macrophages), known to activate the NLRP3 inflammasome in M0 THP-1 macrophages, to mimic a state of a persistent inflammatory response (e.g.; in chronic wounds). After glucose treatment M1, M2 markers, and inflammatory cytokines were quantified. As shown in Fig. 1A-B, untreated macrophages had increased expression of CD86 in a glucose dose dependent manner (Fig. 1A) vs. untreated cells. Concomitantly, HG treatments increased the expressions and the release of pro-inflammatory cytokines (i.e.; IL-1beta and IFNα, Fig. 1C, Additional Fig. 1E) without modulation of the immune-suppressive IL-10 cytokine (Fig. 1C, not deected by ELISA). The concomitant increase of TLR4 and NFκB expressions further validate the pro-inflammatory effects of HG on untreated macrophages (Fig. 1D-E, [40]). We validated that these HG effects were independent of the osmotic stress as 30mM mannitol did not reproduce these data (Additional Fig. 1G).

**Fig. 2:**
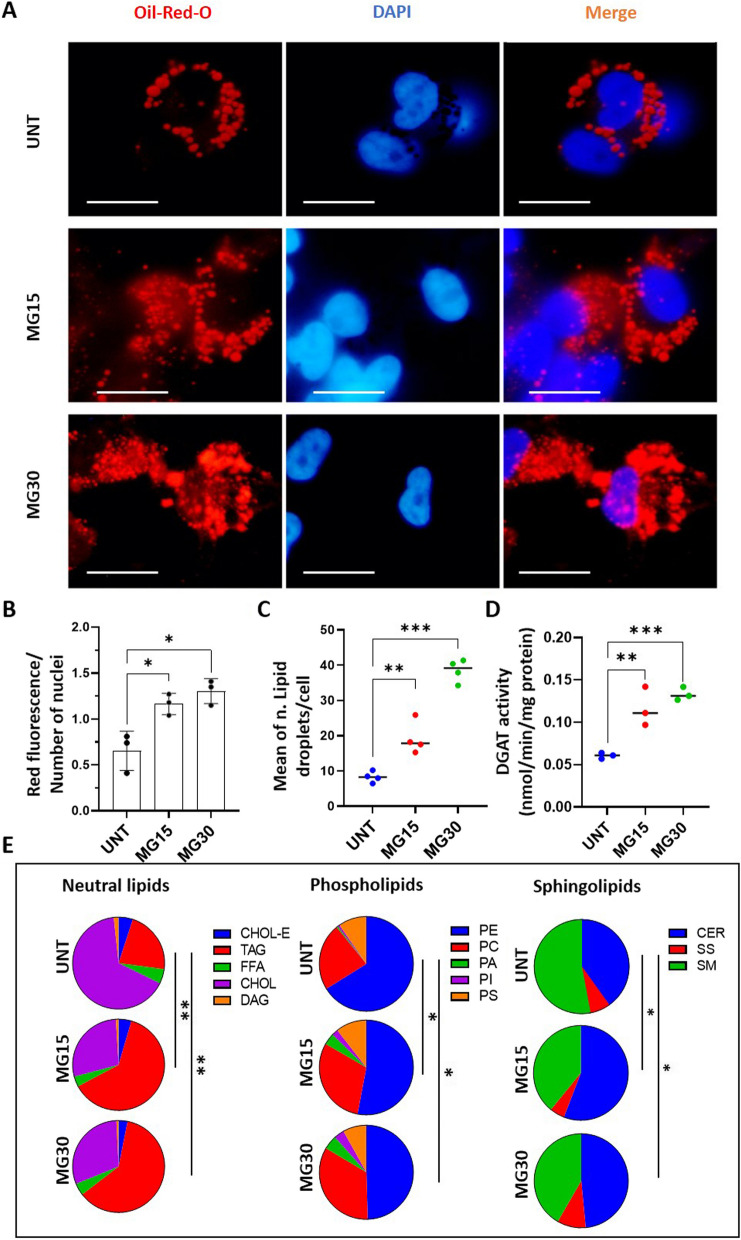
High-glucose concentrations modified macrophage lipid distribution. **A **Oil-red oil and DAPI staining of untreated macrophages (UNT) and MG15 and MG30 macrophages (scale bar=10mm). **B **Quantification of fluorescence intensity normalized to the number of nuclei. Three 3 images were taken per replicate and each dot in the graph is the mean these 3 image counts. **C** Number of lipid droplets per cells (*n* = 4), determined according to [32]. **D** Quantification of DGAT activity by using radio-labelled substrated. **E** Distribution of neutral lipids, phospholipids, and sphingolipids in untreated macrophages (UNT), and in MG15 and MG30 macrophages. Data are expressed as % of total lipids. Significantly different lipid distributions were identified with *chi*-squared tests. CHOL-E: cholesterol ester; TAG: triacylglycerol; FFA: free fatty acid; CHOL: cholesterol; DAG: diacylglycerol; PE: phosphatidylethanolamine; PC: phosphatidylcholine; PA: phosphatidic acid; PI: phosphatidylinositol; PS: phosphatidylserine; CER: ceramide; SS: sphingosine; SM: sphingomyelin; DGAT: diacylglycerol acyltransferase

It is known that macrophage phenotype is strongly associated with modulations of their metabolism [26]. Therefore, to determine how HG might impact the release of lipid-derived EVs, we determine how HG treatments affected macrophage metabolism. Concomitantly with an increase in the hexokinase activity (Additional Fig. 2A), MG30 macrophages had an increased concentration of lactate in the conditioned medium (Fig. 1F) indicating an increase of glycolysis, consistent with their more pronounced M1 phenotype [41]. Conversely, MG15 macrophages did not overproduce lactate suggesting a dose-dependent response of macrophages toward the glucose concentrations. Both MG15 and MG30 macrophages had increased phosphorylation of insulin-induced AKT (Fig. 1G, Additional Fig. 2B). As AKT is a key protein involved in the inhibition of mitochondrial oxidative phosphorylation [42] we suspected that mitochondrial dysfunction may be the underlying factor for increased glycolysis and increased release of lactate in response to HG. Analysis of the mitochondrial activity validated this hypothesis (Additional Fig. 2C). No differences for basal respiration rate (OCR) and mitochondrial and non-mitochondrial oxygen consumption rate were found both in MG15 and MG30 macrophages compared to untreated cells (Additional Fig. 2D-E). However, the coupling efficiency and ATP-related respiration significantly decrease in MG30 cells compared to untreated cells and MG15 (Additional Fig. 2F-G). These results demonstrated an uncoupling effect of the high concentrations of glucose on the ability of mitochondria to utilize the oxidative phosphorylation for ATP synthesis. In agreement, the maximal respiration rate and the amount of extra ATP that can be produced by oxidative phosphorylation in case of a sudden increase in energy demand was significantly reduced in MG30 macrophages (Additional Fig. 2H-I). Consequently, MG15 and MG30 macrophages accumulated huge amount of triacylglycerols (TAG) (Fig. 2A-C) associated with an increase of DGAT activity (Fig. 2D).

Cells can also mobilize TAG to produce precursor FA that are used for phospholipid synthesis (Fig. 1H). In agreement, PA and PI were increased in MG15 and MG30 macrophages (Fig. 2E, Additional Fig. 3A). In addition, HG also modulated the distribution of sphingolipids in macrophages. The ratio of sphingomyelin (SM)/ceramides (CER) was decreased in MG15 and MG30 compared to the untreated macrophages (Fig. 2E, Additional Fig. 3A). This accumulation of ceramides is in line with a previous study demonstrating that M1 macrophages expressed a ceramide-generation-metabolic pattern vs. M2 macrophages [43]. Taken together, these data demonstrate that macrophages adapt their lipid metabolism in response to the increased extracellular concentrations of glucose, resulting in phospholipids remodeling and accumulation of ceramides.

### HG treatments affected the release and the lipid composition of macrophage-derived EVs

**Fig. 3:**
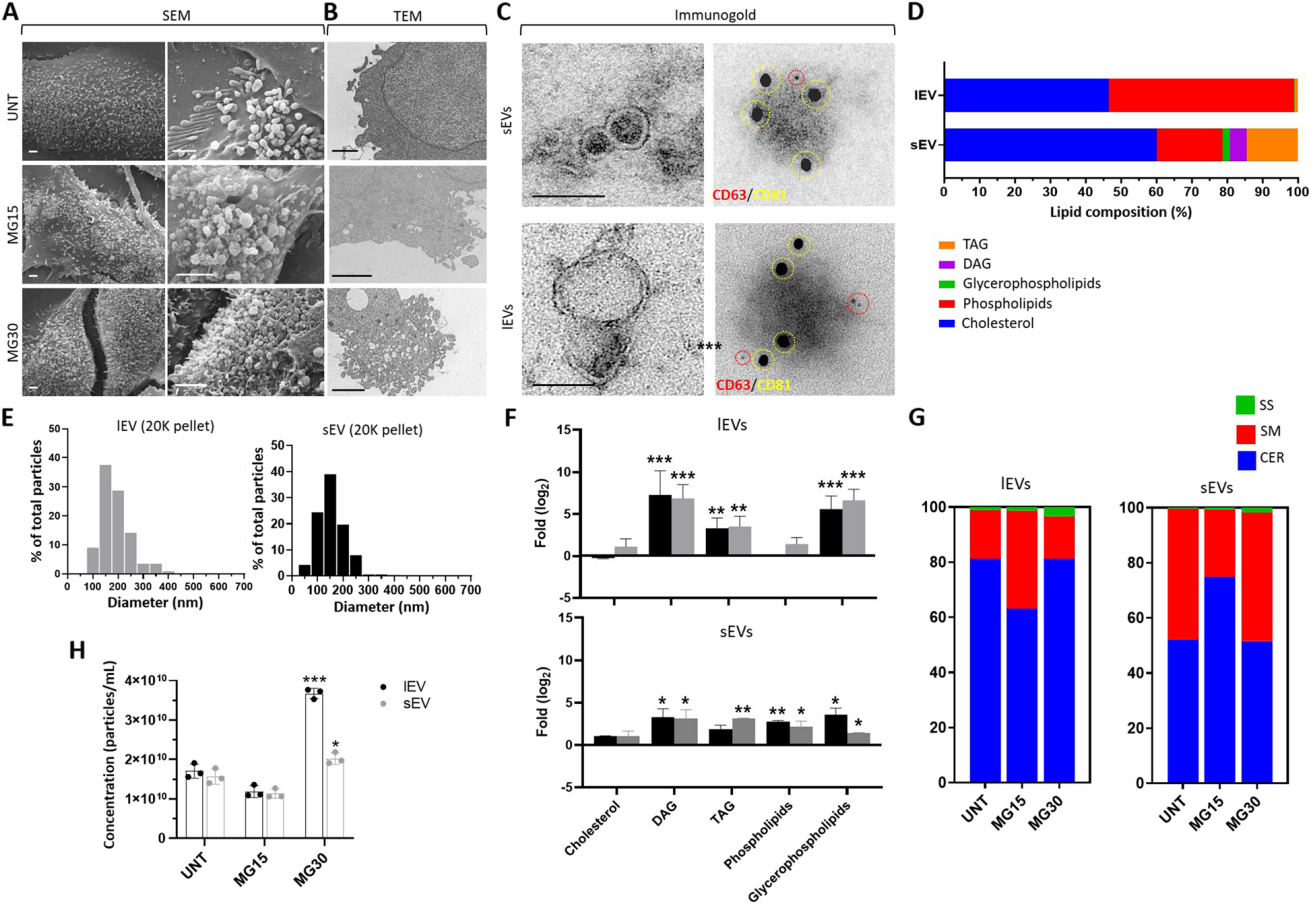
Macrophage-released EV lipid composition and biogenesis are altered by HG treatments.** A **Representative SEM images of the surface of macrophages. **B** Representative TEM images of the membranes of macrophages (scale bar= 500nm). **C **TEM images of lEVs and sEVs released from untreated macrophages (left). **C** right, immunogold labelling of macrophage-derived EVs to detect CD63 and CD81 at the EV surface. Red= CD63, 5nm gold particles, yellow= CD81, 15nm gold particles (scale bar=100 nm). **D** Lipid distribution in lEVs and sEVs from untreated macrophages determined by ^1^H-NMR Spectroscopy. DAG: diacylglycerol; TAG: triacylglycerol. **E** Size distributions of lEVs and sEVs determined by Nanotracking Analyses (NTA) normalized to the total number of detected nanoparticles. **F** Lipid enrichment determined by^1^H-NMR, in lEVs and sEVs released from MG15 and MG30 macrophages compared to untreated macrophages. Values are expressed as log_2_ ratios of untreated macrophages. A representative ^1^H-NMR spectrum obtained at 600 MHz of CD_3_OD/CDCl_3_ lipid extracts of lEVs and sEVs is shown in Additional Fig. 4A-B. **G **Sphingolipid profile of lEVs and sEVs from untreated macrophages, MG15 and MG30 macrophages performed by thin layer chromatography. Representative TLC runs are shown in Additional Fig. 4C. **H** NTA quantification of lEVs and sEVs, expressed as particle/ml, released from untreated macrophages (UNT), MG15 and MG30 macrophages. Values are means ± SD (*n* = 3); *p* values are from student *t*-test (stimulated *vs* untreated), (*)* p* < 0.05, (**) *p* < 0.01, (***) *p*< 0.001. MG15=15 mM glucose, MG30=30 mM glucose

As the HG treatment modifies the concentration of lipids involved in membrane synthesis and curvature [44], we suspected that this may affect the biogenesis and composition of macrophage-released EVs. SEM images revealed numerous buddings on the surface of macrophages (Fig. 3A). However, the plasma membrane of MG30 macrophages produced more buddings compared to MG15 or untreated macrophages (Fig. 3B). Then lEVs and sEVs were purified from fresh conditioned medium (Fig. 3C left, Additional Fig. 3B). Both lEVs and sEVs contained CD81 and CD63 (Fig. 3C right). We were not able to determine firmly the origin of the sEVs which could originate both from small buddings in the plasma membrane and from late endosomal compartments. However, analysis of their lipid distribution by ^1^H-NMR indicated that they had a different origin from lEVs (Fig. 3D, Additional Fig. 4A-B). Compared to lEVs, the sEVs were enriched in cholesterol, glycerophospholipids, TAG, and diacylglycerols (DAG), whereas lEVs were enriched in phospholipids and depleted in cholesterol compared to sEVs. The use of NTA to quantify particle sizes below 500 nm, showed that lEVs were more polydispersed than sEVs (Fig. 3E). In response to HG, MG30 released more EVs (Fig. 3H).

Then we determined the effect of HG treatments on the lipid composition of macrophage-released EVs (Additional Fig. 4C). Figure 3F presents the fold enrichment in each class of lipids vs. lEVs and sEVs released from untreated macrophages, respectively. Both lEVs and sEVs had significant enrichment in TAG/DAG and glycerophospholipids when released from MG15 and MG30 vs. untreated macrophages, and sEVs had higher levels phospholipids. Therefore, the differential lipid enrichment of lEVs and sEVs (Fig. 3D) was lesser pronounced after HG treatments. In cells, the biosynthesis of TAG/DAG and glycerophospholipids begins from the glyceraldehyde 3-P formed *via* glycolysis (Fig. 1H). Thus, the increase of these lipids in HG-treated macrophages-derived lEVs and sEVs reflected the up-regulation of glycoslysis in the producing macrophages. Sphingolipid analyses demonstrated that the SM/CER ratio was lower in lEVs than in sEVs when released from untreated macrophages (Fig. 3G). This differential distribution between the 2 types of EVs has been also observed for other cell types [4], and the quantity of ceramides in EVs seems a way to discriminate between lEVs and sEVs. After treatment with 15mM glucose, sEVs, and lEVs showed opposite modulations of their SM/CER ratios, i.e.; a decrease in lEVs (Fig. 3G) like in the releasing MG15 macrophages (Fig. 2E) but an increase in sEVs. For 30mM glucose, lEVs and sEVs had increased concentrations of sphingosine at the expense of SM, vs. EVs from untreated macrophages (Fig. 3G). Accumulation of sphingosine was also found in the releasing MG30 macrophages (Fig. 3G). Taken together, these data show that after HG treatment, the sphingolipid composition of lEVs reflected the sphingolipid composition of releasing macrophages. This was not the case for sEVs, further supporting that different mechanisms underlie the generation of lEVs and sEVs in macrophages. In addition, glucose treatment affected the release of EVs from macrophages as for 30mM glucose, macrophages released more EVs vs. untreated M0 or MG15 macrophages.

### LEVs and sEVs from HG-macrophages induced markers of M2 macrophages and regulated lipid metabolism of recipient macrophages

**Fig. 4:**
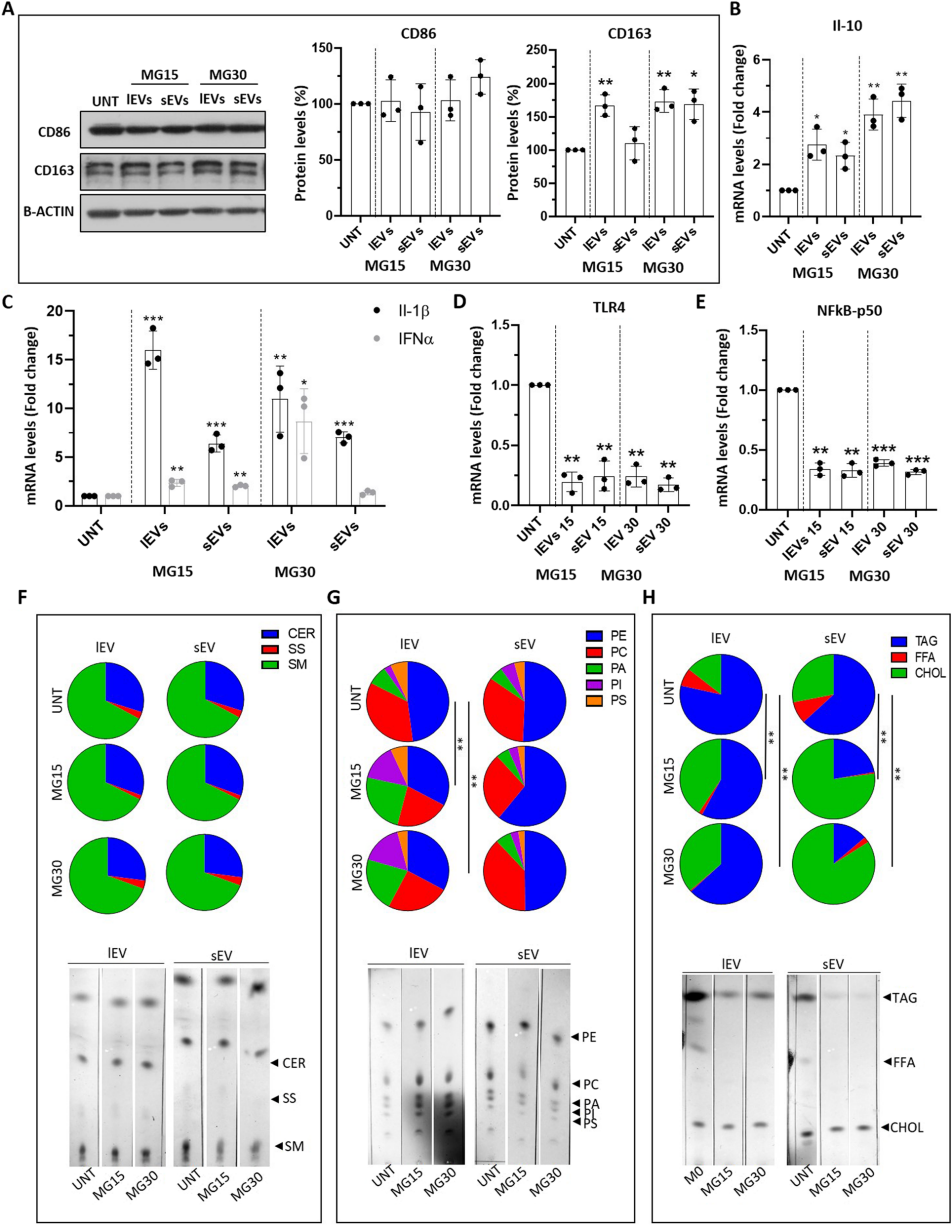
EVs from HG-treated macrophages affect macrophage polarization and lipid composition. **A** Quantification of CD86 and CD163 by WB. mRNA levels of interleukin 10 (Il-10) (**B**), interleukin 1β (Il-1β) interleukin 6 (Il-6), interferon α (INFα) (**C**), toll-like receptor 4 (TLR4) (**D**), and subunit p-50-nuclear factor kappa-light-chain-enhancer of activated B cell (p50-NFkB) (**E**), in macrophages treated with lEVs and sEV released from MG15 and MG30 macrophages. Data are normalized to glyceraldehyde 3-phosphate dehydrogenase (GAPDH) mRNA level and expressed as control. Values are means ± SD (*n* = 3); *p* values are from student *t*-test (EV treated *vs* untreated), (*)* p* < 0.05, (**) *p*< 0.01, (***) *p*< 0.001. (**F**) % of neutral lipids, (**G**) phospholipids and (**H**) sphingolipids in EV-treated macrophages. Significant distributions were identified by a *chi*-squared test. CER: ceramide; SS: sphingosine; SM: sphingomyelin; PE: phosphatidylethanolamine; PC: phosphatidylcholine; PA: phosphatidic acid; PI: phosphatidylinositol; PS: phosphatidylserine; TAG: triacylglycerol; FFA: free fatty acid; CHOL: cholesterol

In vivo, when released from cells, EVs are captured by sentinel macrophages which constantly probe and clean their environment [45–47]. Since HG-treated macrophages released a new population of EVs with altered lipid composition we hypothesized that it could have specific biological functions. Therefore, we treated macrophages with EVs from HG-treated macrophages and analyzed the expressions of polarization markers and cytokines. lEVs and sEVs from MG30 or MG15 macrophages induced the expressions of CD163 (Fig. 4A) and IL-10 (Fig. 4B, Additional Fig. 5A), and decreased the expressions of TLR4 (Fig. 4D) and NFkB-p50 (Fig. 4E) in recipient macrophages. This indicated that EVs from HG-treated macrophages can induce a M2-like phenotype in recipient macrophages. In agreement, recipient macrophages had increased protein levels of the main mitochondrial complexes, compared to untreated macrophages, confirming the metabolic orientation toward an M2 phenotype (Additional Fig. 5B).

Then we analyzed the lipid profile of EV-treated macrophages to determine if EVs affected the lipid metabolism of the recipient macrophages. Neither sEVs nor lEVs from MG15 and MG30 macrophages modified the relative distribution of sphingolipids in recipient macrophages (Fig. 4F). This data indicated it is unlikely that EVs from MG15 and MG30 macrophages transfer sphingolipids (Fig. 3G). The treatment with lEVs, but not sEVs, lead to a decrease in the proportions of phosphatidylcholine (PC) and phosphatidylethanolamine (PE) in recipient macrophages (Fig. 4G). As the decrease of both phospholipids is usually observed in activated macrophages [48], this result supports the effect of macrophage-derived EVs as actors of macrophage activation.

Although LEVs and sEVs from MG15 and MG30 macrophages contained TAG, they induced a reduction in the TAG content of recipient macrophages (Fig. 4H). Therefore, it is unlikely that EVs transfer TAG (Fig. 3F). On the other side, sEVs from MG15 and MG30 macrophages induced much more accumulation of cholesterol inside recipient macrophages than lEVs (Fig. 4H). This in agreement with the higher level of cholesterol in sEVs than lEVs (Fig. 3D-F). These data suggest that sEVs could transfer cholesterol into recipient macrophages.

### EVs from HG-treated macrophages modulate metabolism and secretome of recipient muscle cells

**Fig. 5:**
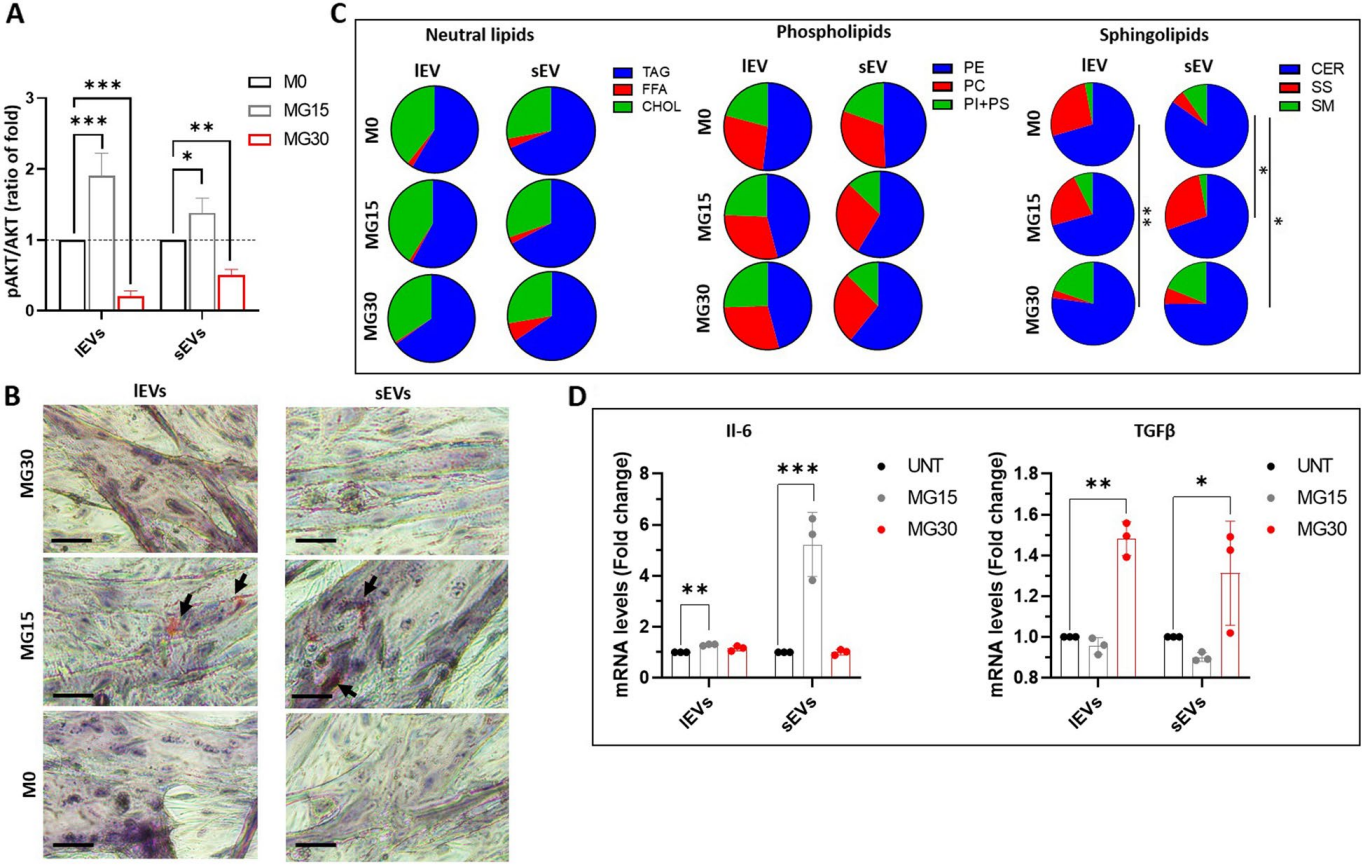
EVs from high glucose-treated macrophages modulate muscle homeostasis. **A** Quantification of insulin-induced phosphorylated AKT in C2C12 myotubes pre-treated with lEVs and sEVs from untreated , MG15, or MG30 macrophages. Data are expressed as ratios (pAKT/AKT)_EV treated_/(pAKT/AKT)_untreated_. Images of the blot are shown in Additional file 5. **B **Lipid droplets detected by Oil-O-Red in C2C12 myotubes pre-treated with lEVs and sEVs from untreated, MG15 and MG30 macrophages (scale bar = 40µm). **C** TLC analyses of neutral lipids, phospholipids, and sphingolipids of C2C12 myotubes treated with lEVs and sEVs from untreated, MG15 and MG30 macrophages. Significant lipid distributions were identified by a *chi*-squared test. TAG: triacylglycerol; FFA: free fatty acid; CHOL: cholesterol; PE: phosphatidylethanolamine; PC: phosphatidylcholine; PI+PS: phosphatidylinositol+phosphatidylserine; CER: ceramide; SS: sphingosine; SM: sphingomyelin. **D** mRNA level of interleukin 6 (Il-6) and (**E**) of transforming growth factor β (TGFβ), in C2C12 myotubes treated with lEVs and sEVs from untreated, MG15, and MG30 macrophages. Data are normalized to TBP mRNA level, then are expressed as fold of controls. Values are means ± SD (*n* = 3); *p* values are from student *t*-test (EV treated *vs* untreated), (*)* p*< 0.05, (**) *p*< 0.01, (***) *p*< 0.001

As EVs from glucose-treated macrophages polarized macrophages into an M2-like phenotype, we wondered whether they could also modulate the metabolism of non-immune cells. In this paper, we have considered skeletal muscle cells (SkM) as recipients. Indeed, in response to hyperglycemia, macrophages infiltrate SkM and release inflammatory cytokines, which can induce muscle wasting and impair muscle function [49]. Therefore, whether EVs from HG-treated macrophages can affect SkM homeostasis similarly to macrophage-released cytokines, is relevant. As shown in Fig. 5A (and Additional Fig. 5C), both lEVs and sEVs from MG15 macrophages induced a hyperphosphorylation of AKT in response to insulin and accumulation of lipid droplets inside recipient SkM cells, already 24 h post-EV treatments (Fig. 5B). Conversely, recipient myotubes treated with lEVs and sEVs from MG30 macrophages strongly reduced insulin sensitivity (Fig. 5A) and lipid storage (Fig. 5B) demonstrating that they had a more pronounced effect on muscle energy balance and fat storage than lEVs and sEVs from MG15 macrophages.

As macrophages treated with MG15- or MG30-derived EVs had altered lipid profiles (Fig. 4F-H) we determined whether muscle treated with macrophage EVs had also altered lipid profiles. Conversely to recipient macrophages (Fig. 4), no significant modifications in phospholipids, neutral lipids, and cholesterol profiles were found in myotubes treated with MG15 or MG30 macrophage-derived EVs (Fig. 5C, Additional Fig. 6). In contrast, lEVs and sEVs from MG30 macrophages induced an increase in SM content in recipient myotubes vs. untreated- or MG15 macrophage-derived EVs (Fig. 5C, Additional Fig. 6). SM is a sphingolipid found in cell membranes, particularly enriched in the plasma membrane. It has been shown that SM interacts with the insulin receptor and thus can affect its activity [50]. If the accumulation of sphingomyelin is excessive, it can lead to the formation of large, rigid lipid rafts, which can hinder membrane protein mobility and signaling [51]. Therefore, the SM increase may partly explain the alteration of insulin sensitivity that we observed in myotubes treated with MG30 macrophage-derived LEVs and sEVs (Fig. 5A).

Finally, we determined whether MG15- or MG30-derived EVs modified the release of myokines from muscle cells. Both lEVs and sEVs from MG15 macrophages induced the expression of Il-6 (Fig. 5D). In addition to its anti-inflammatory function IL-6 has also important regulatory roles by enhancing muscle insulin sensitivity [52]. This might explain why SkM cells treated with lEVs and sEVs from MG15 macrophages have increased insulin sensitivity (Fig. 5A). Conversely, both lEVs and sEVs from MG30 macrophages had a deleterious effect inducing the pro-fibrotic TGFβ expression in recipient SkM cells (Fig. 5E).

## Discussion

Dysregulation of glucose homeostasis results in elevated blood glucose levels, also known as hyperglycemia. It occurs in several metabolic and cardiovascular diseases, sepsis, and cancers and leads to oxidative stress, which in turn promotes inflammation by activating macrophages. Activated macrophages release pro-inflammatory cytokines that can lead to tissue damage and chronic inflammation. The effect of hyperglycemia on the differentiation and activation of subpopulations of macrophages has previously been investigated. It was found that HG-treatment on M1 macrophages pre-stimulated with LPS led to increased secretion of pro-inflammatory cytokines [20, 53], associated with an increase in ROS production and autophagy in macrophages. An HG environment also induced the secretion of pro-inflammatory cytokines during the differentiation of primary human macrophages [54]. In the present study, we show that differentiated M0 macrophages grown in a HG environment expressed pro-inflammatory cytokines demonstrating the direct impact of HG on macrophage polarization into M1. Of note, as THP-1 are from cancerous origin, it is recommaned to grow these cells in RPMI containing 11mM glucose. These growth culture conditions likely explain the small fold changes between untreated cells and cells treated with 15mM glucose sometimes observed.

In parallel, we found that glucose metabolism in M0 macrophages under HG conditions had an increase of glycolysis associated to a mitochondrial dysfunction (more pronounced in 30mM), which had never been described before. This metabolic orientation explains the increased level of CD86, as unlike M2, M1 macrophages have anaerobic metabolism [55]. As M1 macrophages are usually observed in situations of hypoxia (*e.g.*; in adipose tissue of obese patients [56] or during tumor initiation [57]), it was admitted that hypoxia was responsible for the induction M1 macrophages. The results of the present study demonstrate that a HG environment, without hypoxia, also induces a population of pro-inflammatory macrophages through the modulation of their glucose metabolism. Interestingly, the HG environment is not associated with the alteration of insulin-induced AKT phosphorylation in macrophages, such as in metabolic tissues [58]. Consequently, in a diabetic hyperglycemic/hyperinsulinemic environment, AKT activity in macrophages may aggravate the *de novo* lipogenesis and TAG accumulation in macrophages [59].

This study demonstrated that M0 macrophages released a mixed population of sEVs and lEVs, based on their size distributions and morphologies, with very different lipid enrichment, suggesting specific mechanisms or plasma membrane budding locations for their genesis. Both lEVs and sEVs contain cholesterol, but sEVs are more enriched than lEVs. As the technics used for lipid characterization in the producing cells and their released-EVs differ, we were not able to determine whether the differential enrichments in subspecies of phospholipids and sphingosines observed in HG-treated macrophages are reflected on the EV phospholipid and sphingolipid compositions.

Our data show that HG-treated macrophages released EVs enriched in glycerophospholipids and phospholipids, TAG, and DAG, and had a small increase in cholesterol. As EVs are mainly lipid-derived particles, this result indicates that locally at the site of inflammation, the macrophage HG environment is enriched in these lipids. In the body, local accumulation of cholesterol (i.e.; the walls of blood vessels) and TAG (i.e.; adipose tissue) are signals for macrophage recruitment to the site of accumulation. Therefore, the release of lipid-derived EVs which disseminate lipids inside tissues, could be an additional mechanism together with cytokines in the development of chronic inflammation. Indeed, macrophages are phagocytic, and it has been demonstrated that exogenous EVs injected directly into the blood are cleared within two minutes by patrolling monocytes [45] and macrophages [46]. Previous studies have demonstrated that depending on the origin of the EVs, it can result in their polarization into M1 macrophages (*e.g*., adipocyte-derived EVs from diabetic subjects [47]; hepatocyte-derived EVs from NAFLD patients [60]; EVs from M1 macrophages induced by interferon-γ (IFN-γ) and LPS [61] or M2 (Human umbilical cord mesenchymal stem cell derived-EVs [62]; hypoxic glioma cell-derived EVs [63]). Here, we demonstrated that under HG conditions, M1 macrophages released EVs that can polarize recipient macrophages into M2 anti-inflammatory macrophages (*i.e*.; increased oxidative metabolism, increased expressions of CD163 and of the anti-inflammatory Il-10). As the time-course of an inflammatory response involves first the activation of M1 macrophages in the early phase of inflammation, then the activation of M2 macrophages to attenuate inflammation and promote tissue repair, our data demonstrate that macrophage-derived EVs are an intrinsic part of the inflammatory response. In agreement with this study, it was recently demonstrated in a model of endometriosis in mice that M1 macrophage-derived EVs could repolarize macrophages into M2, therefore inhibiting the development of endometriosis [64].

Hyperglycemia is common to many pathologies. Therefore, depending on the biological context the production of M2 macrophages from M1 macrophages through the EV route could be deleterious (*e.g*.; development of chronic inflammation or cancer) or could speed the process of tissue regeneration. In this latter case, it is known that after an injury early M1 macrophage infiltration participates in the clearance of necrotic debris, whereas M2 macrophage infiltration appears later to sustain tissue healing. Now, it is not known whether the two different macrophage populations result from a shift in macrophage polarization or from the recruitment of new monocytes. The data from this study and from [64] indicate that macrophage-derived EVs can shift the phenotype of recipient macrophages.

As SkM is an important tissue for the regulation of glycemia, we determined the effect of EVs derived from M1 macrophages grown in HG on SkM cell homeostasis. MG15-derived EVs induced AKT hyper phosphorylation in response to insulin. Excessive phosphorylation of AKT can result in its prolonged activation and insulin-resistance, as cells become less and less responsive to insulin [65]. As activated AKT stimulates the synthesis of lipids such as TAG, by promoting the uptake of fatty acids and their conversion into storage forms, it explains the accumulation of TAG in response to MG15-derived EVs *vs*. untreated muscle cells. In addition to muscle cell homeostasis, MG15-derived EVs also modulate SkM secretome by inducing ll-6 expression. IL-6 is produced by skeletal muscle as a result of stress, exercise, or injury. It has a protective effect on skeletal muscle (*e.g.*; involved in muscle regeneration or glucose uptake) but also acts as a signaling molecule between muscle and immune cells for muscle regeneration and adaptation [66].

Conversely, EVs derived from macrophages treated with a higher concentration of glucose (30mM) completely altered insulin-induced AKT phosphorylation and TAG storage in recipient SkM cells indicating that macrophage-derived EVs produced in a HG environment may participate in the development of insulin-resistance associated with hyperglycemia [67]. In addition, MG30-derived EVs induced TGFβ expression in recipient muscle cells. TGFβ has a deleterious effect on muscle cells as it induces fibrosis [68]. Therefore, these data demonstrate that EVs released by macrophages have an action on the muscle cell in relation to the glucose concentration used to treat the macrophages. During moderate hyperglycemia, macrophage-derived EVs may contribute to the maintenance of muscle homeostasis, but during more severe hyperglycemia, they would participate in the development of muscle insulin-resistance and the alteration of muscle mass.

## Conclusions

**Fig. 6:**
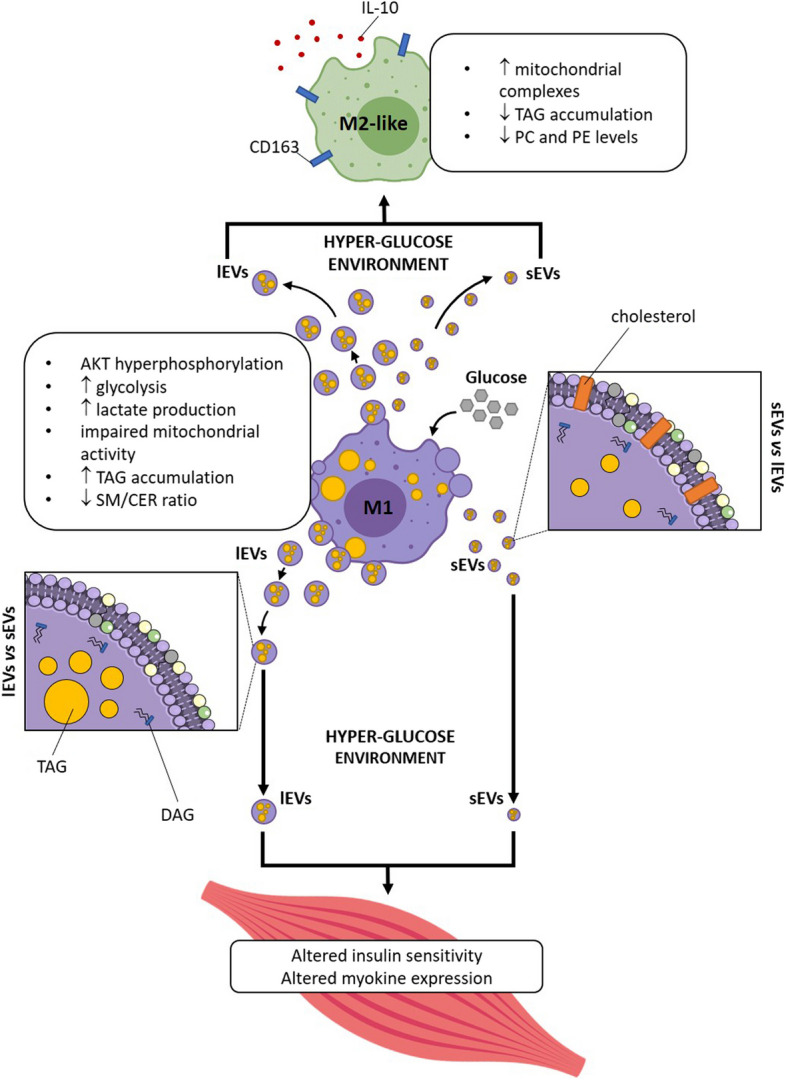
Graphical summary of the main results of this study

This study demonstrates that not only a HG environment shapes macrophage phenotype by regulating their metabolism, but also the biological activity of the EVs released by these macrophages. Surprisingly, EVs derived from M1 in a HG environment induce M2 macrophages which can mitigate inflammatory response. Therefore, in a context of chronic inflammation, these M1-derived EVs could have an unsuspected role in the development of skeletal muscle insulin-resistance associated with diabetes (Fig. 6).

### Supplementary Information

**Additional file 1:** **Figure S1.** (A) MTT assay of THP-1 macrophages grown in RPMI medium 11mM glucose or in EV-depleted medium, for 24h. (B) Quantity of contaminant particles remained in EV-depleted medium detected by Microfluidic Resistive Pulse Sensing (MRPS) as in [28, 29]. (C) WB of the EV-depleted medium or the RPMI medium to detect APOB. (D) MTT assay of THP-1 macrophages grown in RPMI, or in RPMI 15 mM or 30 mM glucose. Values are expressed as % of untreated cells. (E) and (F), ELISA quantification of cytokines in the conditioned medium of macrophages.(G) WB protein quantification of the polarization markers CD163 (M2) and CD86 (M1) in macrophages treated either with mannitol 30mM (osmolarity control) or with glucose 30mM. Values are normalized to beta-actin and expressed as % untreated macrophages.

**Additional file 2: Figure S2.** (A) Images of WB used to quantify phosphorylated AKT (pAKT) in response to insulin and total AKT, in untreated, MG15 and MG30 macrophages. (B) Quantification of Hexokinase activity. (C) Respiratory profiles (OCR) (C), basal respiration (D), mitochondrial and non-mitochondrial oxygen consumption (E), coupling efficiency and ATP-linked respiration (F-G), maximal respiration rate (H) and spare respiratory capacity (I) of untreated, MG15 and MG30 macrophages. Values are means ± SD (*n *= 3); *p* values are from student t-test (treated vs untreated), (*) *p*< 0.05, (**) *p*< 0.01, (***) *p*< 0.001.

**Additional file 3: Figure S3.** (A) Representative TLC profile of neutral lipids, phospholipids, and sphingolipids in untreated, MG15 and MG30 macrophages. (B) TEM images of lEVs and sEVs from untreated macrophages.

**Additional file 4: Figure S4.** (A) Representative ^1^H NMR spectra obtained at 600 MHz of CD3OD/CDCl3 lipid extracts of lEVs and sEVs. (B) Chemical shift and assignments of main peaks in the ^1^H-NMR spectra of CDCl_3_/CD_3_OD extracts of sEVs and lEVs. (C) Sphingolipids TLC analysis of lEVs and sEVs from untreated, MG15 and MG30 macrophages. CER: ceramide; SS: sphingosine; SM: sphingomyelin.

**Additional file 5: Figure S5.** (A) ELISA quantification of interleukin 10 (Il-10) in the conditioned medium of untreated and EV-treated macrophages. (B) Quantification of complexes III, IV and V of the mitochondrial respiratory chain by WB. Data were normalized to total protein levels (Amido Black staining) and expressed as % of untreated macrophages. Values are means ± SD (*n *= 3); *p* values are from student *t*-test (EV-treated *vs* untreated), (*)* p*< 0.05, (**)*p*< 0.01, (***) *p*< 0.001. (B) WB images pAKT/AKT with or without insulin stimulation in C2C12 myotubes treated with lEVs and sEVs from untreated, MG15 and MG30 macrophages.

**Additional file 6: Figure S6.** (A) Representative TLC profile of neutral lipids, polar lipids and sphingolipids of C2C12 myotubes treated with lEVs and sEVs from untreated, MG15 and MG30 macrophages. TAG: triacylglycerol; FFA: free fatty acid; CHOL: cholesterol; PE: phosphatidylethanolamine; PC: phosphatidylcholine; PI+PS: phosphatidylinositol+phosphatidylserine; CER: ceramide; SS: sphingosine; SM: sphingomyelin.

**Additional file 7: Figure S7.** Original WB images used to draw Fig. 1, Additional Fig. 1 and Additional Fig. 2.

**Additional file 8: Figure S8.** Original WB images used to draw Additional Fig. 5.

**Additional file 9: Table S1.** (A) List of antibodies and probes used for Western Blot and Flow cytometry. (B) List of primers used for Real-Time PCR. H: human; M: mouse; R: rat.

## Data Availability

All data from this article are included in this paper.

## Abbreviations

^1^H-NMR: ^1^H-nuclear magnetic resonance
AKT: Protein kinase B
CD: Cluster of differentiation
CER: Ceramide
CHOL-E: Cholesterol ester
CHOL: Cholesterol
DAG: Diacylglycerol
DGAT: Diacylglycerol acyltransferase1/2
EVs: Extracellular vesicles
FA: Fatty acids
FBS: Fetal bovine serum
FFA: Free fatty acid
GLUT1: Glucose transporter 1
HG: High-glucose
IFN: Interferon
IL: Interleukin
ISEV: International society for extracellular vesicles
lEVs: Large extracellular vesicles
LPA: Lysophosphatidic acid
LPS: Lipopolysaccharide
MTT: 3-[4,5-dimethylthiazol-2-yl]-2,5 diphenyl tetrazolium bromide
NTA: Nano-tracking analysis
p50-NFkB: Subunit p50 of the nuclear factor kappa-light-chain-enhancer of activated B cells
PA: Phosphatidic acid
PBS: Phosphate saline buffer
PC: Phosphatidylcholine
PE: Phosphatidylethanolamine
PI: Phosphatidylinositol
PM: Plasma membrane
PMA: Phorbol 12-myristate 13-acetate
PS: Phosphatidylserine
ROS: Reactive oxygen species
RPMI: Roswell Park Memorial Institute medium
RT: Room temperature
sEVs: Small extracellular vesicles
SkM: Skeletal muscle
SM: Sphingomyelin
SS: Sphingosine
TAG: Triacylglycerol
TLC: Thin layer chromatography
TLR4: Toll-like receptor 4
