# Supplementary figures and images for: M1-derived extracellular vesicles polarize recipient macrophages into M2-like macrophages and alter skeletal muscle homeostasis in a hyper-glucose environment
Source: Cell Commun Signal. 2024 Mar 27;22:193. doi: 10.1186/s12964-024-01560-7 (PMC10967050; doi:10.1186/s12964-024-01560-7)

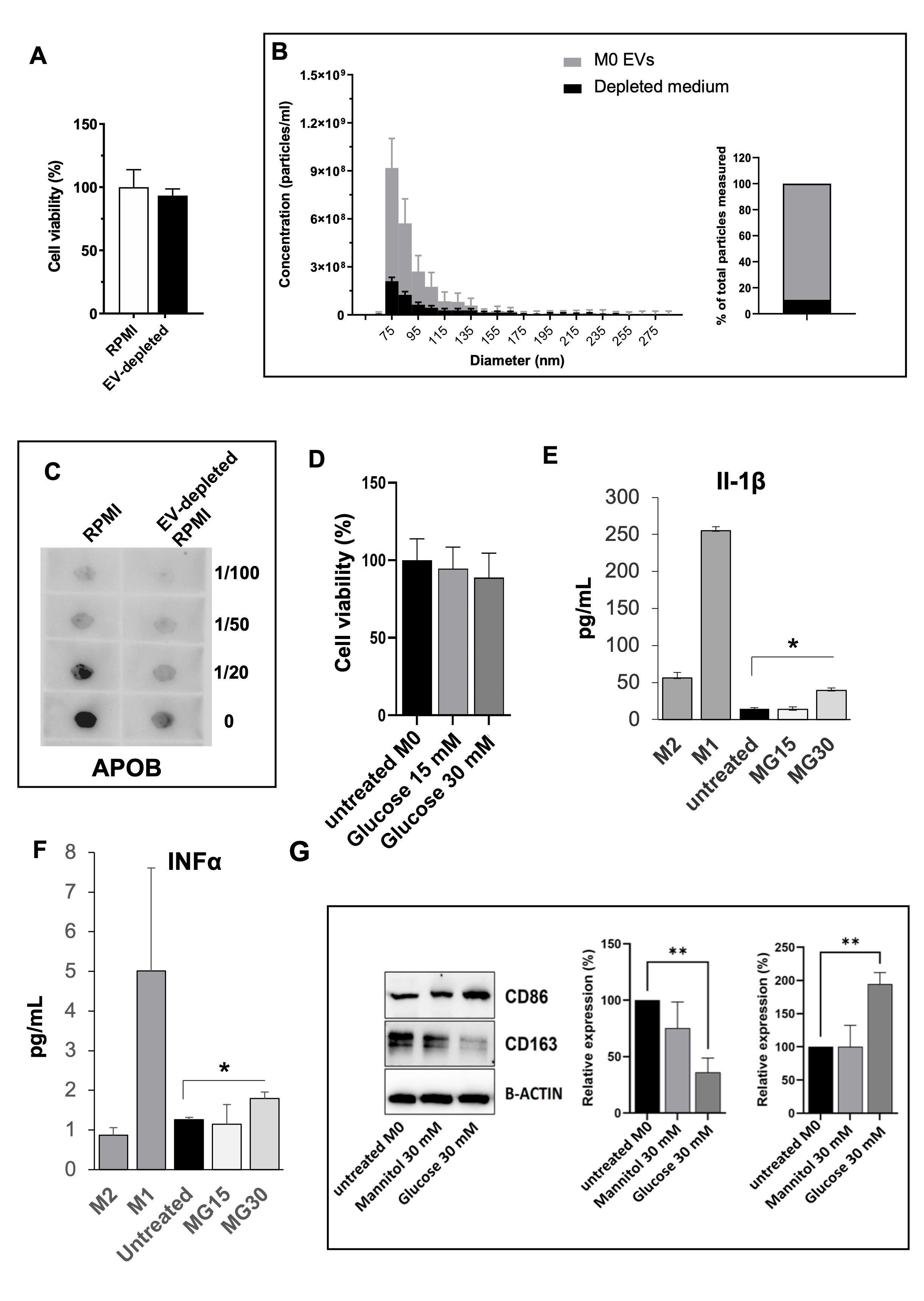

Supplement: Supplementary file 1 — Additional file 1: Figure S1. (A) MTT assay of THP-1 macrophages grown in RPMI medium 11mM glucose or in EV-depleted medium, for 24h. (B) Quantity of contaminant particles remained in EV-depleted medium detected by Microfluidic Resistive Pulse Sensing (MRPS) as in [28, 29]. (C) WB of the EV-depleted medium or the RPMI medium to detect APOB. (D) MTT assay of THP-1 macrophages grown in RPMI, or in RPMI 15 mM or 30 mM glucose. Values are expressed as % of untreated cells. (E) and (F), ELISA quantification of cytokines in the conditioned medium of macrophages.(G) WB protein quantification of the polarization markers CD163 (M2) and CD86 (M1) in macrophages treated either with mannitol 30mM (osmolarity control) or with glucose 30mM. Values are normalized to beta-actin and expressed as % untreated macrophages. [file 12964_2024_1560_MOESM1_ESM.jpg]

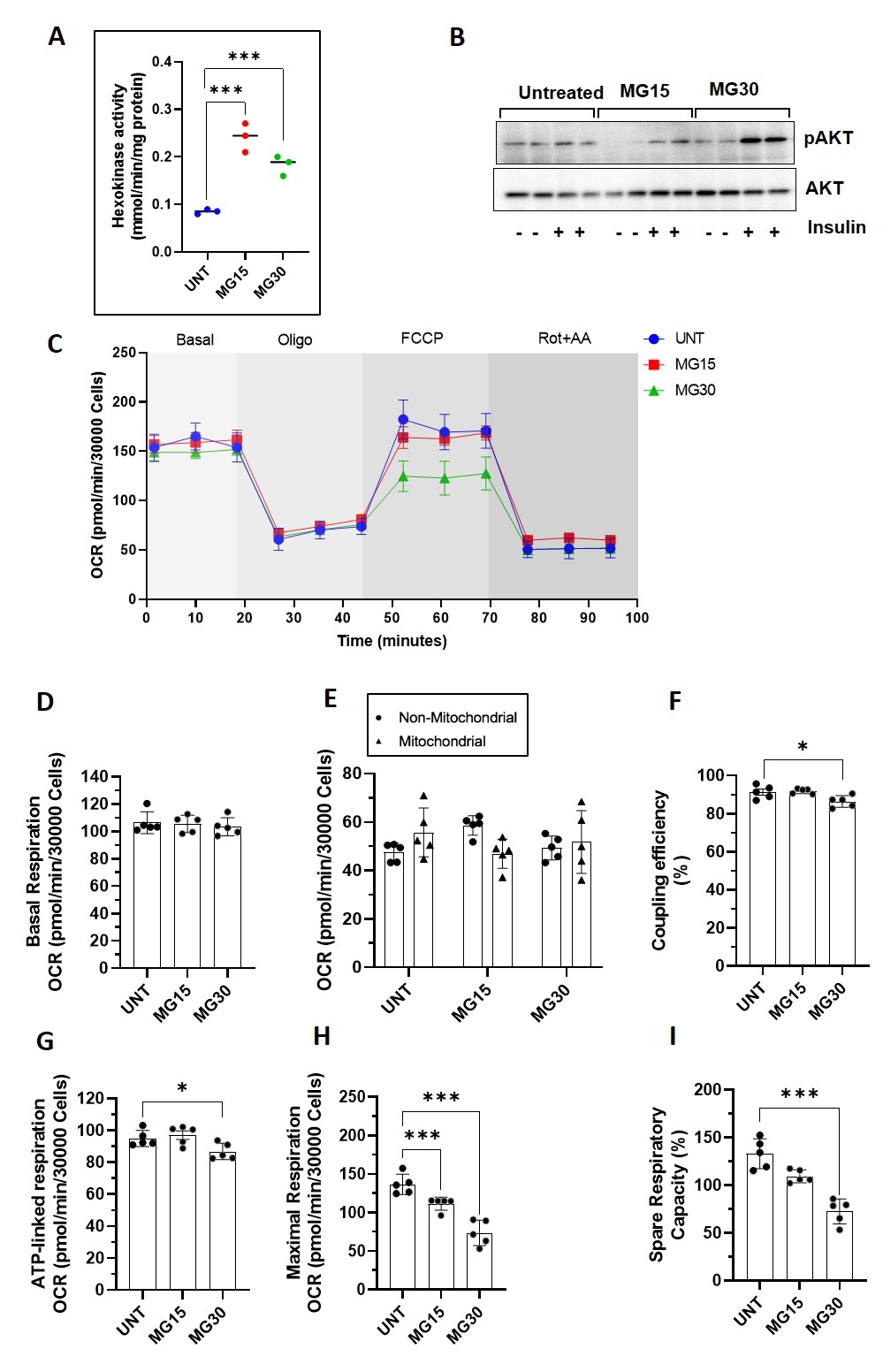

Supplement: Supplementary file 2 — Additional file 2: Figure S2. (A) Images of WB used to quantify phosphorylated AKT (pAKT) in response to insulin and total AKT, in untreated, MG15 and MG30 macrophages. (B) Quantification of Hexokinase activity. (C) Respiratory profiles (OCR) (C), basal respiration (D), mitochondrial and non-mitochondrial oxygen consumption (E), coupling efficiency and ATP-linked respiration (F-G), maximal respiration rate (H) and spare respiratory capacity (I) of untreated, MG15 and MG30 macrophages. Values are means ± SD (n = 3); p values are from student t-test (treated vs untreated), (*) p< 0.05, (**) p< 0.01, (***) p< 0.001. [file 12964_2024_1560_MOESM2_ESM.jpg]

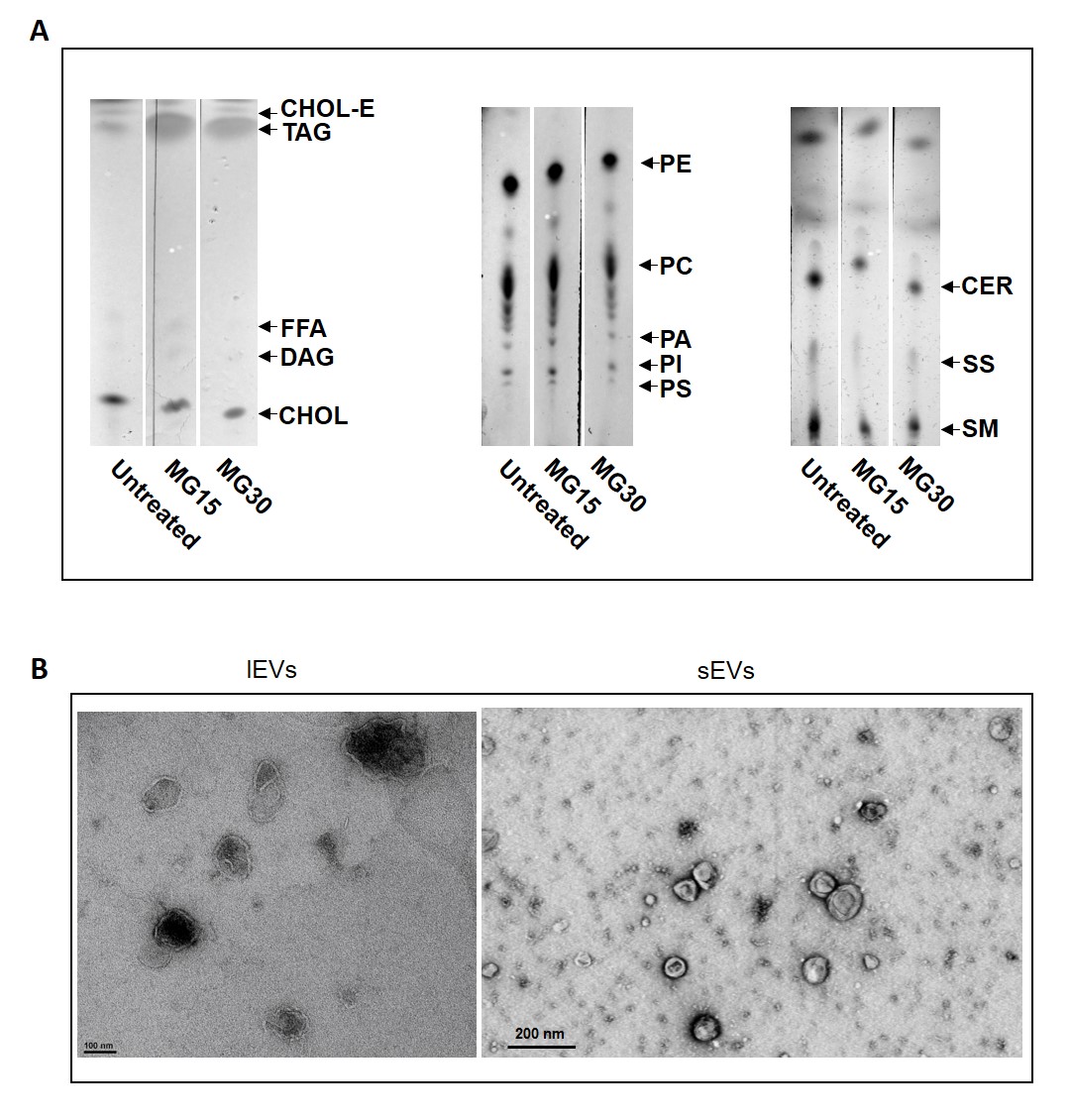

Supplement: Supplementary file 3 — Additional file 3: Figure S3. (A) Representative TLC profile of neutral lipids, phospholipids, and sphingolipids in untreated, MG15 and MG30 macrophages. (B) TEM images of lEVs and sEVs from untreated macrophages. [file 12964_2024_1560_MOESM3_ESM.jpg]

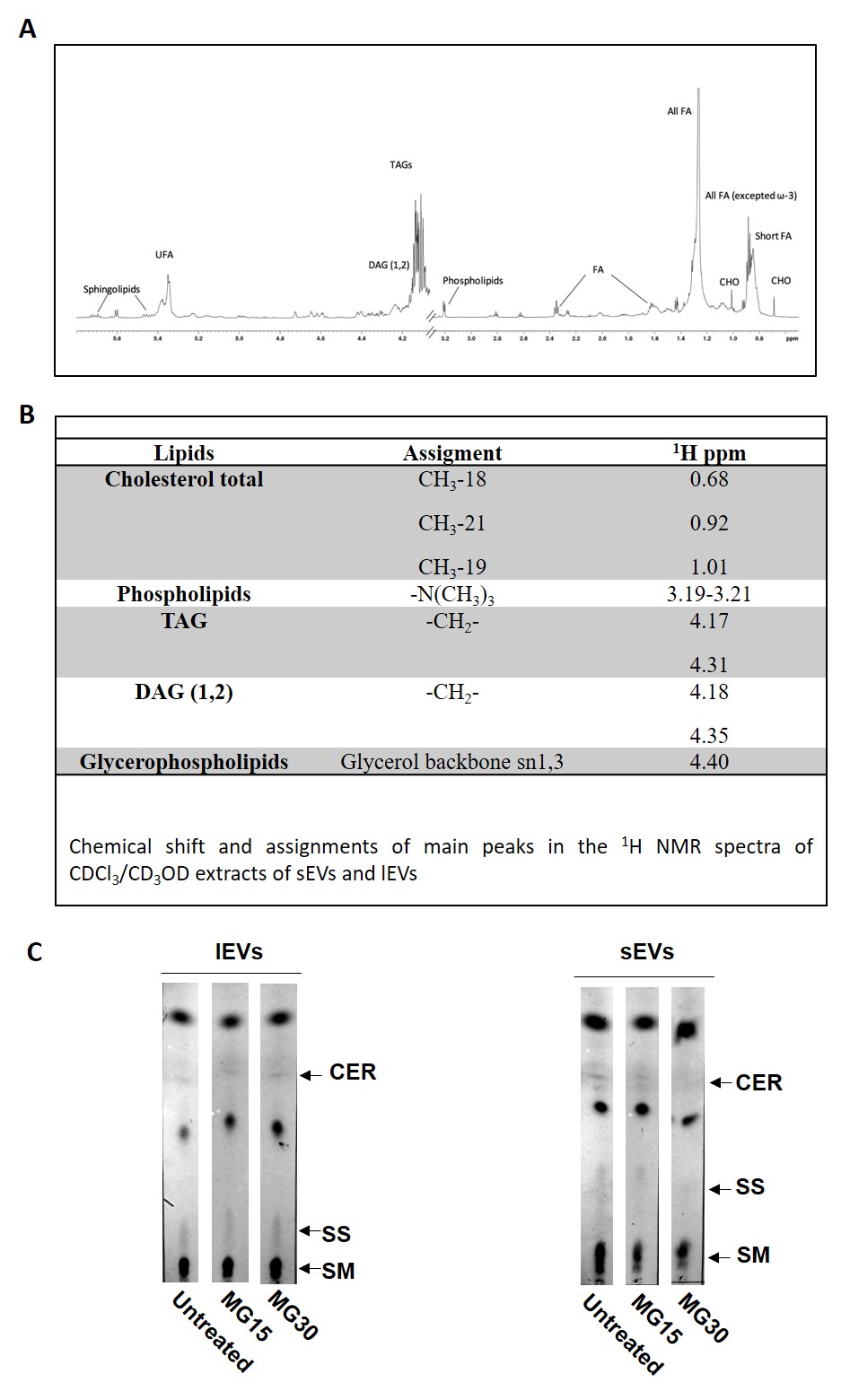

Supplement: Supplementary file 4 — Additional file 4: Figure S4. (A) Representative 1H NMR spectra obtained at 600 MHz of CD3OD/CDCl3 lipid extracts of lEVs and sEVs. (B) Chemical shift and assignments of main peaks in the 1H-NMR spectra of CDCl3/CD3OD extracts of sEVs and lEVs. (C) Sphingolipids TLC analysis of lEVs and sEVs from untreated, MG15 and MG30 macrophages. CER: ceramide; SS: sphingosine; SM: sphingomyelin. [file 12964_2024_1560_MOESM4_ESM.jpg]

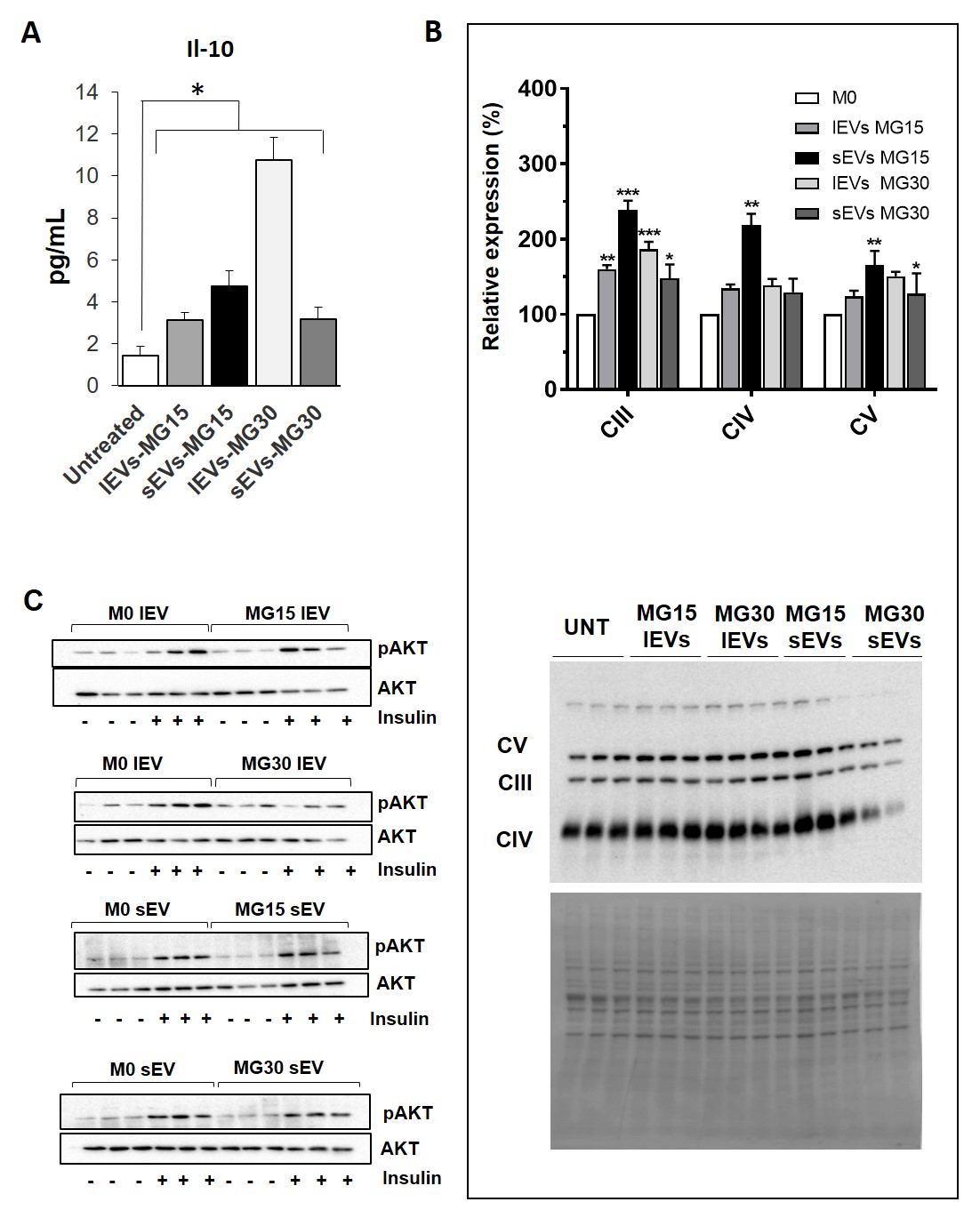

Supplement: Supplementary file 5 — Additional file 5: Figure S5. (A) ELISA quantification of interleukin 10 (Il-10) in the conditioned medium of untreated and EV-treated macrophages. (B) Quantification of complexes III, IV and V of the mitochondrial respiratory chain by WB. Data were normalized to total protein levels (Amido Black staining) and expressed as % of untreated macrophages. Values are means ± SD (n = 3); p values are from student t-test (EV-treated vs untreated), (*) p< 0.05, (**)p< 0.01, (***) p< 0.001. (B) WB images pAKT/AKT with or without insulin stimulation in C2C12 myotubes treated with lEVs and sEVs from untreated, MG15 and MG30 macrophages. [file 12964_2024_1560_MOESM5_ESM.jpg]

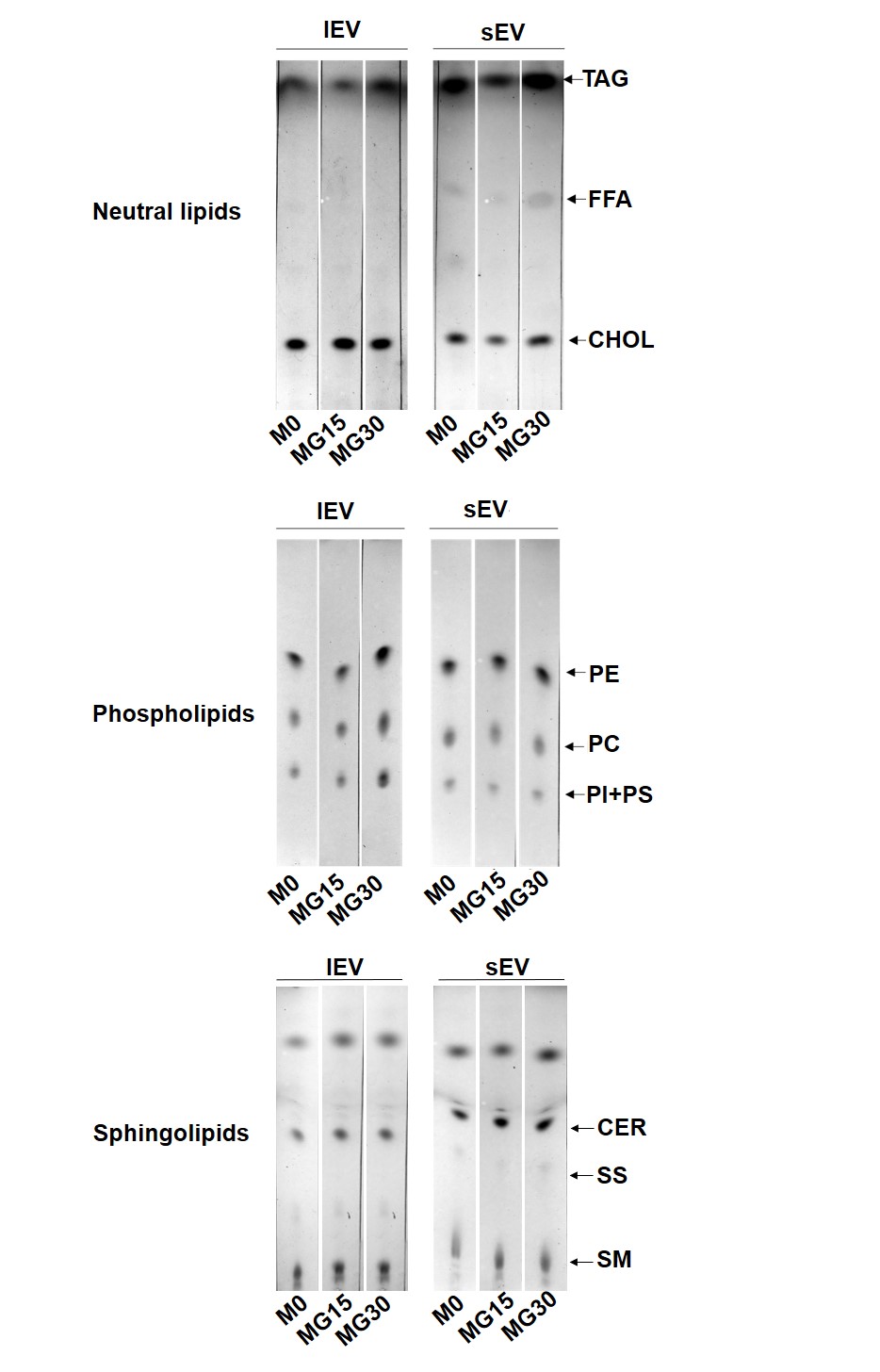

Supplement: Supplementary file 6 — Additional file 6: Figure S6. (A) Representative TLC profile of neutral lipids, polar lipids and sphingolipids of C2C12 myotubes treated with lEVs and sEVs from untreated, MG15 and MG30 macrophages. TAG: triacylglycerol; FFA: free fatty acid; CHOL: cholesterol; PE: phosphatidylethanolamine; PC: phosphatidylcholine; PI+PS: phosphatidylinositol+phosphatidylserine; CER: ceramide; SS: sphingosine; SM: sphingomyelin. [file 12964_2024_1560_MOESM6_ESM.jpg]

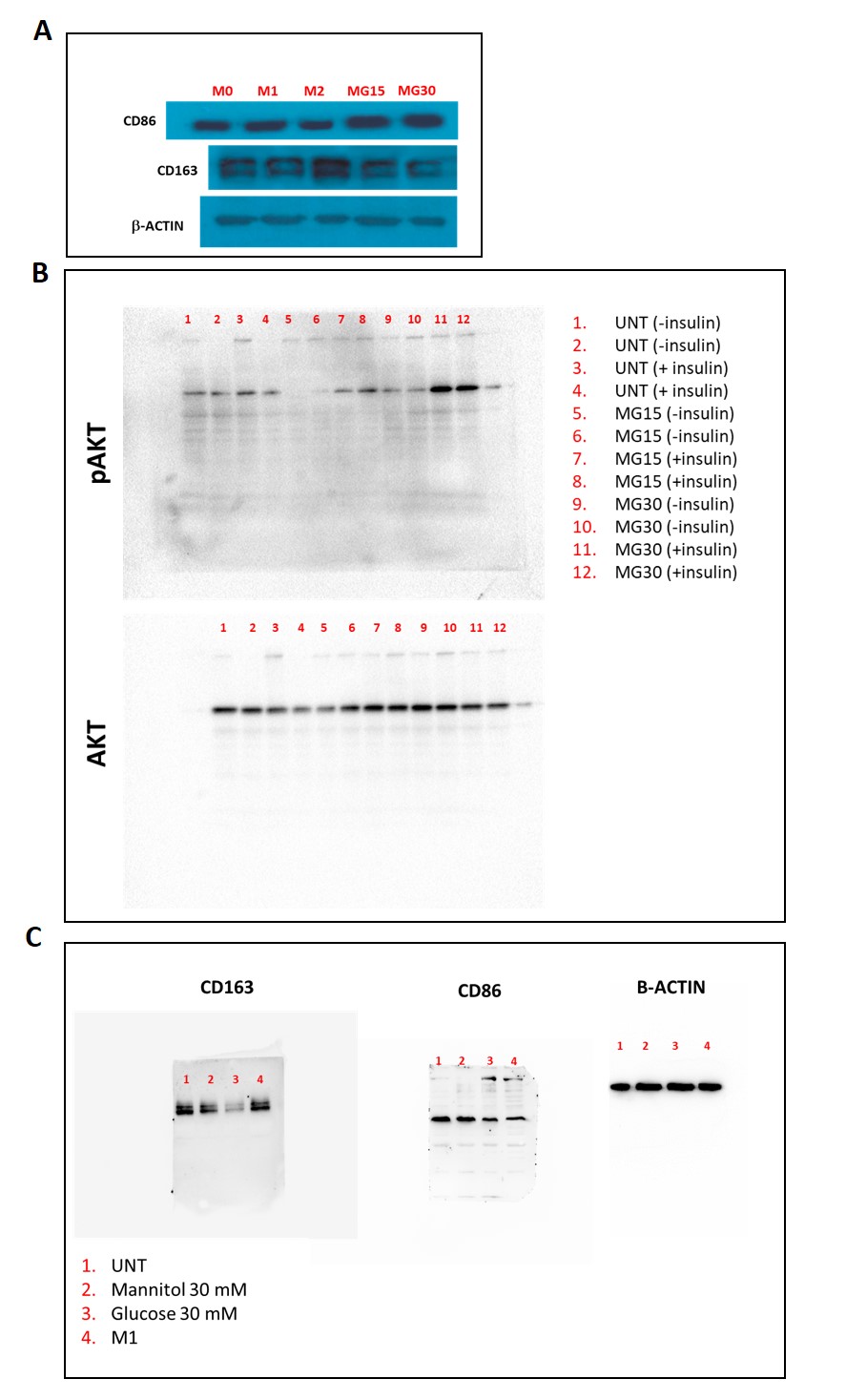

Supplement: Supplementary file 7 — Additional file 7: Figure S7. Original WB images used to draw Fig. 1, Additional Fig. 1 and Additional Fig. 2. [file 12964_2024_1560_MOESM7_ESM.jpg]

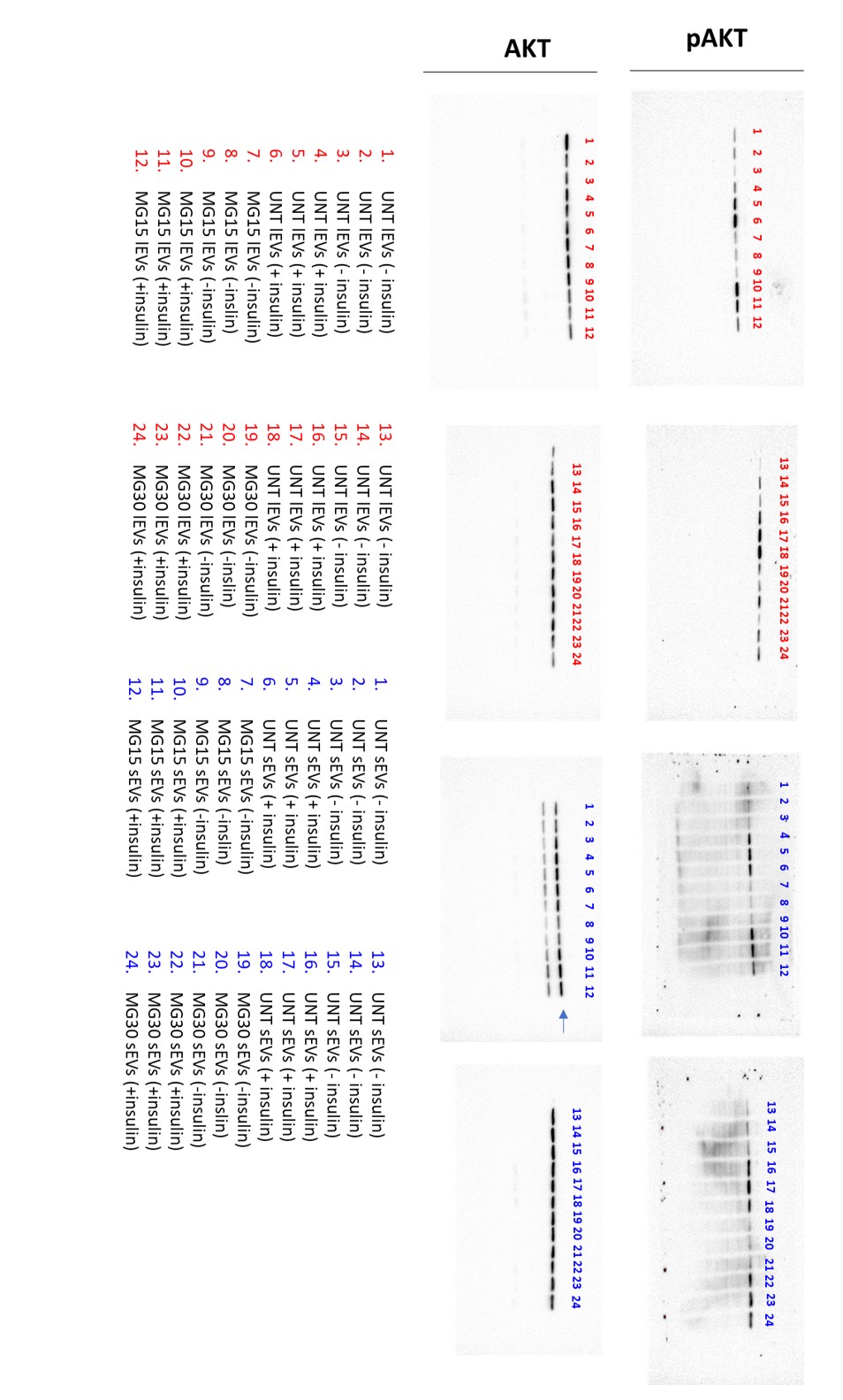

Supplement: Supplementary file 8 — Additional file 8: Figure S8. Original WB images used to draw Additional Fig. 5. [file 12964_2024_1560_MOESM8_ESM.jpg]

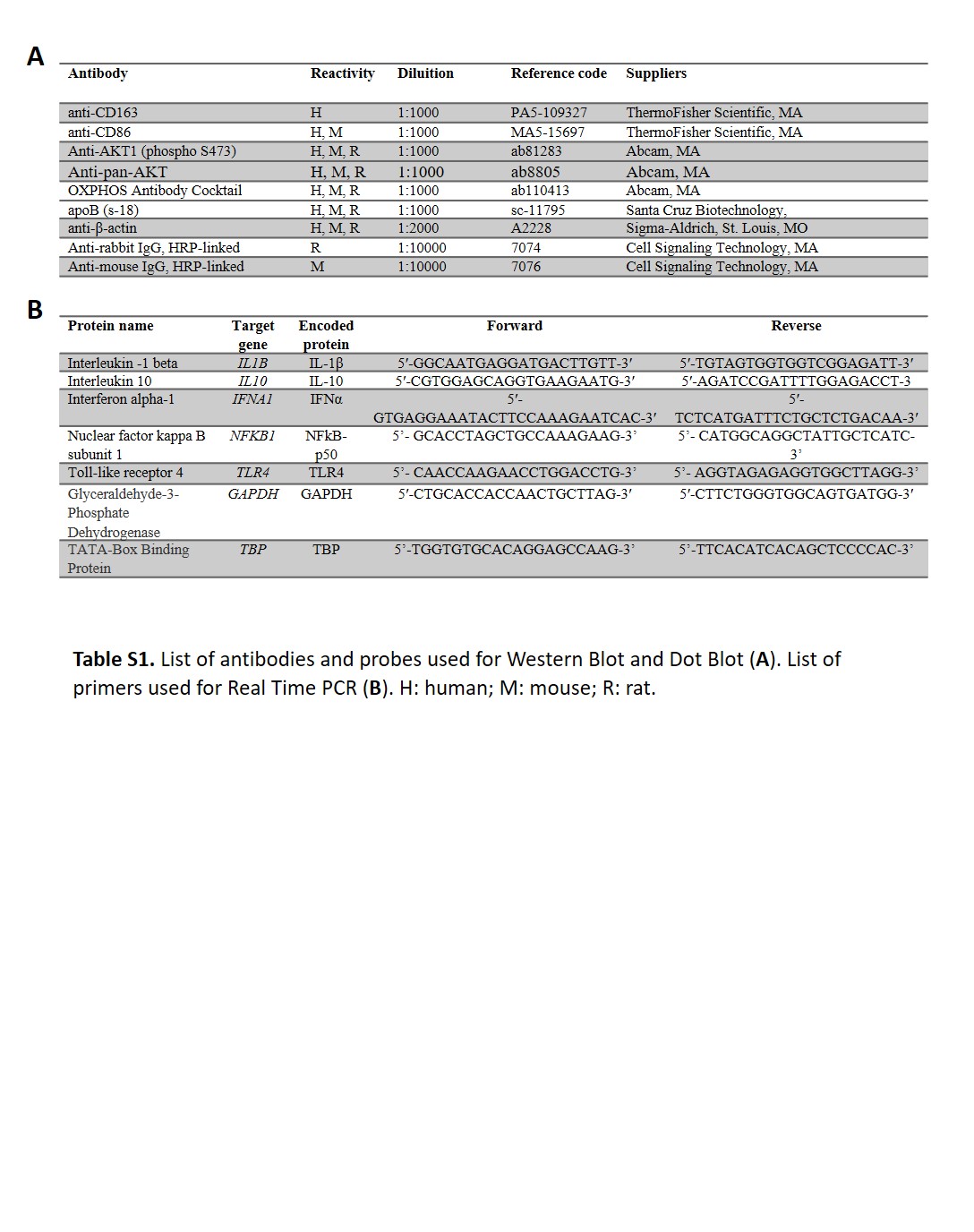

Supplement: Supplementary file 9 — Additional file 9: Table S1. (A) List of antibodies and probes used for Western Blot and Flow cytometry. (B) List of primers used for Real-Time PCR. H: human; M: mouse; R: rat. [file 12964_2024_1560_MOESM9_ESM.jpg]
